# Barriers to functional connectivity across contrasting landscapes in the widespread but declining Iberian common toad

**DOI:** 10.1038/s41598-026-36452-y

**Published:** 2026-02-19

**Authors:** Carlos Caballero-Díaz, Gregorio Sánchez-Montes, Pedro Tarroso, Irene Castrosín, Íñigo Martínez-Solano

**Affiliations:** 1 Departamento de Biología, Universidad Autónoma de Madrid, C/ Francisco Tomás y Valiente, 7, 28049 Madrid, Spain; 2 Asociación Herpetológica Española (AHE), Apdo. Correos 191, 28911 Leganés, Madrid, Spain; 3 Present Address: Departamento de Biodiversidad y Biología Evolutiva, Museo Nacional de Ciencias Naturales (MNCN-CSIC), c/ José Gutiérrez Abascal, 2, 28006 Madrid, Spain; 4 CIBIO, Centro de Investigação em Biodiversidade e Recursos Genéticos, InBIO Laboratório Associado, Universidade do Porto, Campus de Vairão, 4485-661 Vairão, Portugal; 5 BIOPOLIS Program in Genomics, Biodiversity and Land Planning, CIBIO, Campus de Vairão, 4485-661 Vairão, Portugal

**Keywords:** Amphibians, *Bufo spinosus*, Ecological barriers and corridors, Gene flow, Land uses, Landscape resistance, Ecological genetics, Population genetics, Herpetology, Conservation biology

## Abstract

Understanding how topographical and environmental features influence population genetic structure is pivotal in ecology, evolution and conservation. Landscape genetics allows to perform such inference, along with identification of ecological corridors and barriers to dispersal. Yet, results are often constrained by the spatial scale of analysis, making generalization difficult, especially for widespread species with broad ecological niches. Comparative assessments of landscape connectivity in heterogeneous study areas can help identify elements with general impacts on population connectivity as well as idiosyncratic features with leverage only on certain types of habitats, providing key information for the design of context-dependent conservation measures. We assessed functional connectivity in a widespread but declining amphibian, the Iberian Common Toad (*Bufo spinosus*) in two areas of central Spain with contrasting landscape features: the Guadarrama foothills and the Alcarria plateau. We genotyped > 500 individuals from 33 sites with 11 microsatellites and characterized genetic diversity and structure in both areas. We conducted landscape genetic analyses to reveal the differential importance of landscape features in population connectivity across study areas. We found consistent patterns, with temporary streams and moderate slopes facilitating gene flow, while major rivers and highways acted as barriers, especially in the Alcarria plateau, where population structure was more pronounced. Land cover variables partly explained patterns of functional connectivity, but their effects varied across regions, with different land classes facilitating or disrupting gene flow. Our results underscore the importance of comparative landscape genetics studies for the design of tailored conservation measures in widespread species.

**Supplementary Information:**

The online version contains supplementary material available at 10.1038/s41598-026-36452-y.

## Introduction

Understanding how topographical and environmental features influence the genetic structure of populations is pivotal in ecology, evolution and conservation^1–3^. As a fundamental approach, landscape genetics aims to quantify the effect of landscape variables and their spatial configuration on the distribution of genetic variation, identifying potential corridors for species as well as barriers to gene flow^4,5^. However, generalization of the results of landscape genetics studies is often limited by the spatial scale of analysis^6,7^. This is particularly constraining when studying widespread species occupying a broad range of habitats, because focusing on a particular study area does not account for the full breadth of their ecological preferences and can misrepresent conservation challenges^8,9^. While comparative studies across broad spatial or ecological ranges are ideal, they are generally unfeasible, but regional comparisons across contrasting landscapes can also provide valuable insights into connectivity patterns in widespread species. Replicating landscapes with shared and different features across regions can help uncover both general drivers of connectivity and landscape-specific responses^8,10–12^. From a conservation perspective, species with restricted ranges or habitat specialists often receive more attention^13^, but considering common, generalist species in landscape genetics studies is important because the negative effects of population fragmentation and isolation are often unnoticed across their larger ranges^14^.

Amphibians are frequently used as model systems in landscape genetics studies, because they tend to present spatially structured populations at small spatial scales^15–17^ and are strongly affected by habitat loss and fragmentation^18,19^. Pond-breeding amphibians show high fidelity to aquatic breeding sites, but also undertake dispersal events contributing to gene flow across the landscape matrix^16,20,21^. However, human activities—such as urbanization, industrialization, or intensive agriculture—have extensively modified natural landscapes^22,23^, with negative impacts on the aquatic and terrestrial ecosystems where amphibians complete their biphasic life cycle^24,25^. Habitat degradation and loss affect patterns of structural connectivity (i.e., the physical relationships between habitat patches^26,27^), causing population fragmentation, favouring isolation and potentially leading to local extinctions^6,28^. In disturbed areas, functional connectivity (i.e., how effectively individuals move through the landscape matrix^27,29^) is compromised^30,31^, with negative effects for the viability of amphibian populations^32^. In the current context of increasing impacts of human activities on ecosystems, it is thus important to understand the relationships between landscape elements and functional connectivity in amphibians^15,33–35^. The combined use of genetic markers and environmental layers allows inference of population structure and potential barriers to gene flow, and can be applied to identify areas where management efforts can be directed towards promoting connectivity^17,33,34^.

Different landscape elements have been shown to shape population structure, dispersal and functional connectivity in amphibians^36,37^. These include both natural and anthropogenic features, like mountains, which often represent strong barriers to dispersal^38,39^; water courses, which width and water flow influence population structure^40,41^; agriculture, which restricts or promotes connectivity in intensive^42^ or traditional^10,35^ crop plots, respectively; vegetation cover, with contrasting effects on dispersal among species^43^; and urbanization, which generally impedes connectivity^39,44^. Due to the limited dispersal capacity of amphibians^20^, topography and environmental variables have a major role in connectivity patterns across species^34,38,44^. However, most landscape genetic studies typically assess small portions of the ranges of target species, providing relevant information for the management of local populations and potential insights on the role of similar landscape elements in other regions. This is a limiting factor in the study of geographically widespread taxa, in which the conservation status of populations can strongly differ across their distribution range^45,46^. In these cases, full coverage of the ecological niche of the species cannot generally be achieved, but comparative studies on replicated landscapes can still provide valuable insights, with applications in conservation.

**Fig. 1:**
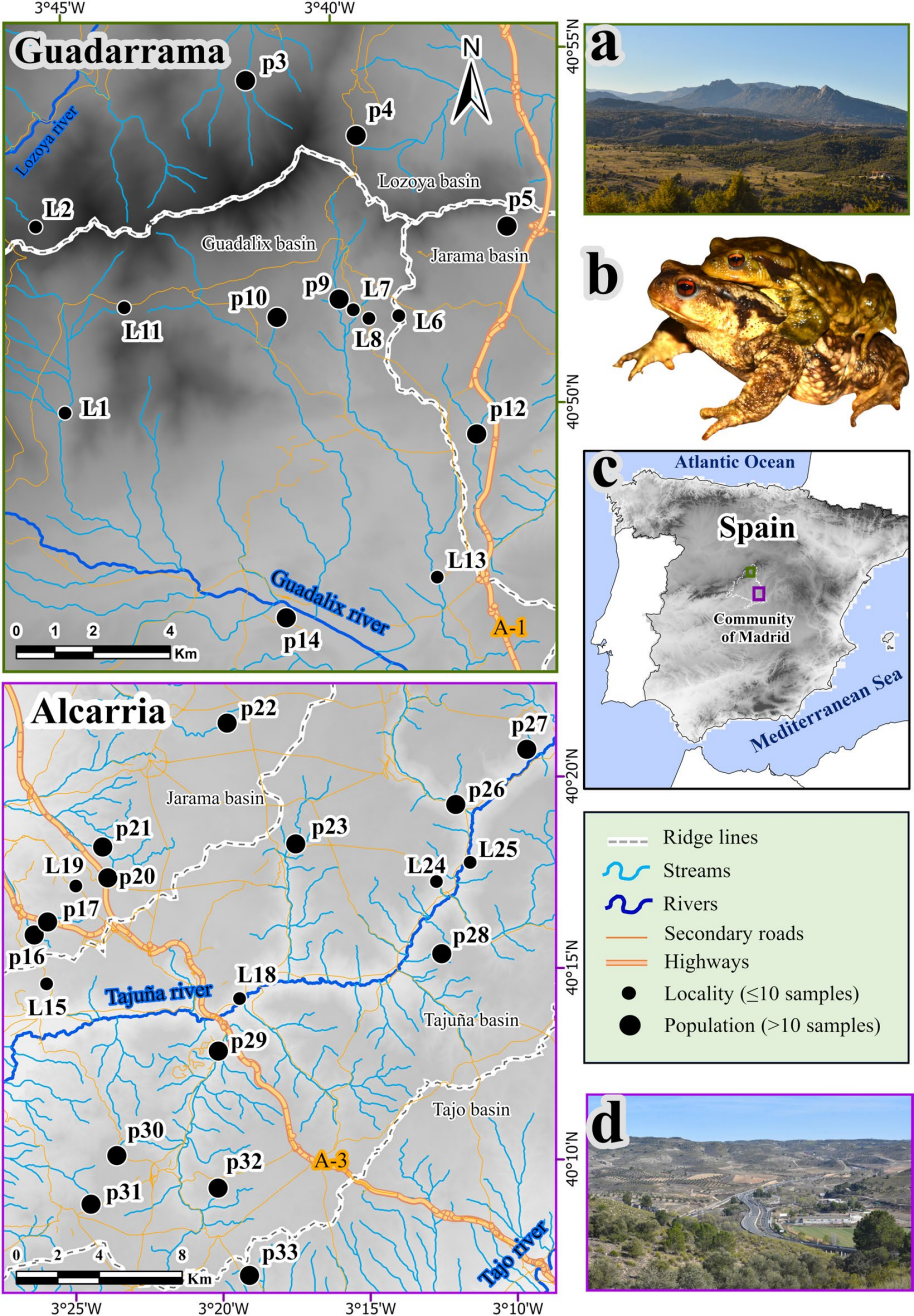
Maps of the two study areas (Guadarrama, in green; Alcarria, in purple), with sampling populations (“p”; >10 genotyped individuals) and localities (“L”; ≤ 10 genotypes). Photos of representative landscapes (a = Guadarrama, d = Alcarria), and their location in the Iberian Peninsula (c) are also shown. The background colour (grey) represents elevation range. b = mating pair of the target species, *Bufo spinosus*. Maps were built with ArcGis Pro 3.3.0 (https://www.esri.com/es-es/arcgis/products/arcgis-pro/overview).

The Iberian common toad, *Bufo spinosus* Daudin 1803 (Fig. 1d), is a widespread, generalist species distributed across southwestern Europe and north Africa^47^. They are medium-to-large-sized toads undertaking seasonal mass migrations from foraging and refugial terrestrial areas to their breeding sites^48^. In the Iberian Peninsula, the species occupies a broad range of habitats, from sea level to mountainous areas surpassing 2,500 m above sea level (m.a.s.l)^49^. While populations are still geographically widespread in Iberia, especially in the north, where they remain abundant, the species has experienced significant declines in the central and southern two-thirds of Spain^48,49^, although the causes of this negative trend are not fully understood. In this context, comparative landscape genetics studies encompassing topographically and ecologically distinct study areas can document patterns of genetic diversity and structure across different types of habitats, and provide insights on the role of different landscape elements in population connectivity.

Here, we conducted such a comparative study, focusing on two contrasting landscapes (regarding topography and main land uses) in Central Spain to explore the role of environmental and anthropogenic features in promoting or disrupting connectivity among populations of the widespread Iberian common toad (*B. spinosus*). We assessed functional connectivity among toad populations in two study areas in the Community of Madrid: the sierra de Guadarrama foothills, where the species is a common inhabitant of long hydroperiod streams, ponds, and water reservoirs, with a largely continuous distribution, and the Alcarria plateau, where populations are fragmented and suitable breeding sites are scarce. Populations in the Guadarrama mountains occupy humid, mixed or densely vegetated habitats more akin to those used by the species in the northern half of Spain, where they are abundant, especially across forested mountain habitats. In contrast, populations in the Alcarria plateau, which inhabit moorlands and valleys at lower altitudes in the region, are scarcer and representative of the declining populations in eastern and southern Spain, which live in areas with drier conditions, low availability of water bodies and reduced vegetation cover^39,48^. We genotyped over 500 individuals from 33 geographical locations with 11 microsatellites to characterize and compare patterns of genetic diversity and population structure in both study areas. Then, we conducted landscape genetic analyses to investigate which factors have a stronger leverage on functional connectivity among populations of *B. spinosus* in each region. Specifically, we tested five non-mutually exclusive hypotheses pertaining to the relative effects of rivers, roads, land use, ridge lines and slope on patterns of genetic structure. Considering the species abundance and distribution in the region^48^ and because of the environmental characteristics of both study areas, similar to northern (Guadarrama) or eastern and southern Spain (Alcarria), where the species is respectively relatively abundant and continuously distributed or scarce and fragmented, we expect higher functional connectivity in the Guadarrama mountains than in the Alcarria. We discuss implications of our results for the conservation of the species in central and southern Spain, where it is in slow but constant decline.

## Methods

### Study areas and sampling

The study was conducted in two areas with contrasting landscapes in the Community of Madrid (central Spain), separated ∼60 km (Fig. 1). The first (Guadarrama) is located in the foothills of the Guadarrama mountains, a hilly landscape in the north of the region, whereas the second (Alcarria) is part of the Alcarria plateau, a flat, heterogeneous landscape largely comprising crops, moorlands and valleys. The characteristics of each study area are summarized in Table 1.

**Table 1:** Summary of the main biotic and abiotic features characterising each study area (Guadarrama and Alcarria).

	Guadarrama	Alcarria
Surface	223.3 km^2^	867.2 km^2^
Elevation Av (min- max)	1,045 m.a.s.l. (743–1,823)	714 m.a.s.l. (521–863)
Human population density	78 inhabitants/km^2^	122 inhabitants/km^2^
Precipitation (annual average)	471.9 mm	430 mm
Temperature (anual average)	11.3 °C	13.8 °C
Soil	Silicicolous materials, predominantly gneiss and granites	Limestone and gypsum, including sands and clays in the Tajuña river basin
Water courses	Guadalix river (permanent hydroperiod), Albalá, Jóbalo streams (temporary)	Tajuña river (permanent hydroperiod), de la Veguilla, del Valle streams (temporary)
Main activities	Livestock farming and forestry	Agriculture (wheat, corn, olive, vineyards)
Land uses	Forests (32.8%), heathlands and shrublands (17.6%), meadows and pastures (14.9%), agricultural areas (10.8%), urban areas (6.4%), and combined vegetation (6.2%)	Agricultural areas (61.1%), forests (13.3%), heathlands and scrublands (8.5%), combined vegetation (7.1%) and urban areas (5.4%)
Vegetation	Pyrenean oak woodlands (*Quercus pyrenaica*), holm oak (*Quercus ilex*) and ash (*Fraxinus angustifolia)* “dehesas”, pine forests (*Pinus sylvestris*), and Mediterranean shrubland (*Quercus ilex*, *Cistus ladanifer*, *Retama sphaerocarpa*, *Thymus* spp., *Rosmarinus officinalis*, *Cytisus scoparius*)	Mediterranean “maquis” (*Quercus ilex*,* Quercus coccifera*) and calcicolous scrublands (*Thymus vulgaris*,* Genista scorpius*,* Fumana thymifolia*,* Lavandula stoechas*,* Stipa tenacissima*), and Aleppo pine plantations (*Pinus halepensis*)
Roads	Several secondary roads	Major highway A-3 (northwest to southeast), numerous secondary roads

Nocturnal surveys at both study areas were conducted from February to April 2022, which comprises the breeding season of *B. spinosus* in the region^48^. In both study areas, we visited all water bodies where the species is known to occur and sampled all major breeding populations, i.e., excluding those where the species is present at low abundances or the actual breeding site is unknown^48,50,51^. Because of the high philopatry of the species and the geographic distances among sampling sites, largely beyond maximum recorded dispersal distances for the species (around 2.5 km, Sánchez-Montes and Martínez-Solano, unpublished data), all or most adult individuals sampled are expected to have originated at those sites rather than representing dispersers from other sites. To maximize sampling success, nocturnal sampling session with temperatures above 4 °C and rainfall or high relative humidity were conducted throughout the breeding season. During each survey, a group of 2–3 people searched for adult individuals, examining the aquatic breeding areas and their immediate terrestrial surroundings during ∼1 h per sampling site. We targeted adult individuals instead of tadpoles or juveniles to minimize the probability of sampling closely related individuals, which could bias population genetic inferences^52^. Individuals were captured by hand or using dip nets. We clipped 3 phalanges of the 4th toe of the right hind limb of each adult *B. spinosus* and stored these tissue samples individually in vials with absolute ethanol. Animals were immediately released upon processing. We conducted several surveys per site throughout the 2022 breeding season; all the field material used was disinfected with bleach 1:10 to avoid the transmission of pathogens among breeding sites. We collected > 10 individual tissue samples in 7 and 14 breeding areas of *B. spinosus* of the Guadarrama and Alcarria study areas, respectively (hereafter referred to as sampling populations, Fig. 1; Table 2 and Supplementary Table S1). At some additional sites, ≤ 10 tissue samples were obtained to complement our sampling (hereafter, referred to as localities, Fig. 1 and Supplementary Table S1).

**Table 2:** Genetic diversity estimates at each sampling population (*N* > 10).

Site	Sampling population	Sa	*N*	Na (SE)	Ho (SE)	He (SE)	F_IS_ (SE)	HW	PA
p3	Garganta de los Montes	G	20	7.45 (0.67)	0.68 (0.07)	0.70 (0.05)	0.19 (0.02)	1	0
p4	Lozoyuela	G	17	5.55 (0.58)	0.68 (0.09)	0.64 (0.06)	0.16 (0.03)	1	0
p5	La Cabrera	G	21	5.91 (0.55)	0.76 (0.06)	0.66 (0.05)	0.14 (0.01)	0	1
p9	Valdemanco 4	G	42	9.09 (0.57)	0.72 (0.03)	0.73 (0.03)	0.17 (0.01)	0	6
p10	Bustarviejo 2	G	40	9.36 (0.62)	0.71 (0.04)	0.76 (0.02)	0.19 (0.01)	1	3
p12	Cabanillas de la Sierra	G	21	6.73 (0.59)	0.73 (0.04)	0.72 (0.03)	0.17 (0.01)	0	0
p14	Guadalix de la Sierra	G	22	8.09 (0.74)	0.74 (0.06)	0.74 (0.04)	0.16 (0.01)	0	2
p16	Arganda del Rey 1	A	31	5.27 (0.38)	0.71 (0.05)	0.61 (0.04)	0.15 (0.01)	2	0
p17	Arganda del Rey 2	A	40	5.82 (0.35)	0.69 (0.04)	0.64 (0.04)	0.16 (0.01)	1	0
p20	Arganda del Rey 4	A	14	6.73 (0.75)	0.82 (0.06)	0.71 (0.05)	0.13 (0.01)	0	2
p21	Arganda del Rey 5	A	22	7.82 (0.62)	0.75 (0.03)	0.76 (0.03)	0.18 (0.02)	0	2
p22	Pozuelo del Rey	A	14	6.09 (0.57)	0.71 (0.06)	0.63 (0.05)	0.15 (0.01)	0	0
p23	Valdilecha	A	22	7.27 (0.67)	0.69 (0.06)	0.70 (0.05)	0.17 (0.02)	0	0
p26	Ambite 1	A	22	8.09 (0.72)	0.75 (0.05)	0.70 (0.05)	0.15 (0.01)	0	3
p27	Ambite 2	A	14	4.09 (0.42)	0.64 (0.09)	0.55 (0.07)	0.16 (0.01)	0	0
p28	Carabaña	A	21	6.36 (0.52)	0.72 (0.04)	0.65 (0.04)	0.16 (0.01)	0	2
p29	Perales de Tajuña	A	20	6.73 (0.39)	0.73 (0.05)	0.70 (0.04)	0.17 (0.01)	0	0
p30	Valdelaguna	A	20	7.00 (0.54)	0.72 (0.05)	0.69 (0.05)	0.17 (0.01)	0	0
p31	Chinchón	A	23	7.64 (0.62)	0.73 (0.04)	0.74 (0.03)	0.18 (0.01)	0	0
p32	Belmonte de Tajo	A	23	7.64 (0.50)	0.75 (0.04)	0.74 (0.03)	0.16 (0.01)	0	0
p33	Villarejo de Salvanés	A	24	7.91 (0.56)	0.75 (0.03)	0.74 (0.03)	0.16 (0.01)	0	2

Site codes and names, study area (Sa, G = Guadarrama, A = Alcarria), sample size (N), average number of alleles per locus (Na), observed heterozygosity (Ho), expected heterozygosity (He), inbreeding coefficient (F_IS_), number of markers with significant deviations from Hardy-Weinberg equilibrium (HW), number of private alleles (PA). Estimates of Na, Ho, He and F_IS_ are accompanied by standard error (SE) values (in parentheses). Site codes as in Fig. 1 and supplementary table S1. The complete genotype matrix is available in supplementary table S9.

### DNA extraction and genotyping

We isolated genomic DNA from tissue samples with commercial kits (DNeasy 96 Blood & Tissue, Qiagen), following the manufacturer’s protocol. For genotyping, we targeted 12 polymorphic microsatellites optimized for *B. spinosus*^53^. Microsatellite amplification was conducted via polymerase chain reaction (PCR), with primers arranged into 3 multiplex reactions (Supplementary Table S2). Multiplex reactions 1 and 2 consisted of: (1) initial denaturation (95 °C, 5 min), (2) 30 cycles of denaturation (95 °C, 30 s), annealing (60 °C, 1.5 min), and extension (72 °C, 30 s), and (3) final extension (60 °C, 30 min). Multiplex 3 reaction was similar, but in the second step the annealing temperature was 58 °C^53^. The sizes of amplified DNA fragments were analysed in an ABI PRISM 3730 sequencer (Applied Biosystems), and the resulting chromatograms were screened with GENEMAPPER v4.0 (Applied Biosystems) to produce the final genotype matrix for downstream analyses.

### Genetic diversity

We conducted population genetic analyses on the 21 sampling populations (7 in Guadarrama, 14 in Alcarria). First, we examined potential departures from Hardy-Weinberg genotypic proportions (HWP) for each locus and population using R package *pegas*^54^, and linkage disequilibrium (LD) between pairs of loci for each sampling population using R package *genepop*^55^. We run tests with 10,000 replicates per sampling population (HWP), and 10,000 initial permutations (dememorization), 1,000 batches and 10,000 iterations per batch in LD tests. We applied the sequential Bonferroni correction to adjust the significance of *p*-values in multiple tests of HWP and LD^56^. We also used R packages *adegenet*^57^ and *pegas* to calculate mean values and the corresponding standard errors for the following indices of genetic diversity per population: allelic richness (mean number of alleles per locus), private alleles (PA; alleles found exclusively in one population), expected heterozygosity (He) and observed heterozygosity (Ho), and the inbreeding coefficient (Wright´s fixation index; F_IS_).

### Genetic structure and population differentiation indices

We characterized the genetic structure of *B. spinosus* across both study areas based on Bayesian clustering analyses with program Structure v2.3.4^58^, using the complete dataset with all genotyped individuals of all sampling sites (including both sampling populations and localities). We did not consider prior information about sampling sites (locprior = 0) and ran 10 replicates for *K* values (representing the number of genetic clusters) ranging from 1 to 15 in Guadarrama and from 1 to 20 in Alcarria (to account for possible substructure within sites). We used an admixture model with correlated allele frequencies, and the number of burn-in and post-burn-in iterations was set to 100,000. We then used Structure Harvester^59^ to assess the relative likelihood of *K* values based on the original method (i.e., increasing likelihood values)^58^ and on the ΔK method^60^, and Clumpak^61^ to summarize clustering results.

In addition, we calculated pairwise genetic differentiation among sampling populations (*N* > 10) within each of the two study areas using the function fastDivPart in R package *diversity*^62^. Specifically, we calculated Hedrick’s G′_ST_ index, which provides a good description of allelic differentiation among populations while informing about population structure at the landscape scale in philopatric organisms^63,64^. We also calculated the differentiation index D_JOST_^65^, which was strongly correlated with G′_ST_ values (Pearson correlation coefficient = 0.955,* p*-value < 0.001; R² = 0.913). Thus, we conducted downstream landscape genetic analyses using only pairwise G′_ST_ values.

Finally, we tested for Isolation by Distance (IBD) in both study areas with Mantel tests, using the complete dataset of genotyped samples and applying the ‘mantel’ function in R package *vegan*^66^.

### Landscape genetic analyses

To assess which landscape elements influence functional connectivity among *B. spinosus* populations in each study area, we used a three-step approach, consisting of: (1) preparing landscape layers, (2) optimizing resistance models, and (3) model evaluation.

We obtained fine scale land use/cover (LUC) data from the high-resolution Spanish Information System of Land Occupation (SIOSE; http://www.siose.es). SIOSE layers are provided at a scale of 1:25,000, characterized by a minimum polygon width of 15 m and thematic resolution of 12.5 m, as well as a minimum mapping unit of 0.5-2 ha that depends on the land cover type (e.g., 0.5 ha for wetlands, 1 ha for urban areas, 2 ha for forests). We reclassified LUC units across study areas for simplicity, following^34^, resulting in these categories: agriculture crops, combined vegetation, forest, heathland and shrubland, meadows and pasture, open soils, transport routes, rocky outcrops, urban and artificial, and water surface (Supplementary Table S3). Additionally, road information was obtained from the National Centre of Geographic Information (CNIG, https://www.centrodedescargas.cnig.es). This dataset provides information on dual carriageways (highways with two or more lanes per direction, accounting for 98.7% of traffic in the region) and two-lane roads (one lane per way, accounting for 1.3% of traffic in the region) (https://www.transportes.gob.es). We also downloaded a watercourse layer from the Environmental Department of the Spanish Government (https://www.miteco.gob.es/es/cartografia-y-sig/ide/descargas/agua/red-hidrografica.html). Rivers and streams in this dataset are ranked according to the Pfafstetter classification, which is based on the topology, length and territorial surface occupied by each watercourse. We reclassified the watercourses in two categories: rivers, with permanent hydroperiod, and streams, with temporary hydroperiod. We also extracted the five main ridge lines of the study areas, i.e., the highest areas separating major river basins. For that, we downloaded spatial data on Spanish watersheds (https://www.miteco.gob.es/es/cartografia-y-sig/ide/descargas/agua/cuencas-y-subcuencas.html) and combined stream basins into three major units in Guadarrama (corresponding to the Guadalix, Lozoya and Jarama river basins) and another three in Alcarria (corresponding to the Tajuña, Jarama and Tajo river basins)—all of them containing at least one genotyped sampling population, see Fig. 1—in order to calculate the perimeter of the basin. Average (av), maximum (max) and minimum (min) elevation values (in m.a.s.l.) were calculated for each line ridge in both study areas: Guadalix/Lozoya (av = 1,591.1, max = 1,830.6, min = 1,294.3 m.a.s.l), Guadalix/Jarama (av = 976.6, max = 1,537.7, min = 846.6 m.a.s.l.), Lozoya/Jarama (av = 1,249.5, max = 1,558.2, min = 1,040.1 m.a.s.l.), Tajuña/Jarama (av = 773.7, max = 833.5, min = 683 m.a.s.l.) and Tajuña/Tajo (av = 775.4, max = 809.3, min = 674.1 m.a.s.l.). Finally, slope information was calculated with the tool “Slope” in Spatial Analyst Tools (ArcGis 10.3.1), using Digital Terrain Model information (DTM, https://www.centrodedescargas.cnig.es) at a resolution of 200 m. Then, we reclassified slope values (%) in four classes: 0–10, 10–20, 20–30, and 30–41%. All layers were rasterized using R package *Terra*^67^.

We conducted an optimization of resistance models for landscape elements based on spatial and genetic data, as detailed below. While our approach shares conceptual similarities with methods like R package *ResistanceGA*^68^, which applies genetic algorithms to optimize resistance surfaces, we conducted a tailored approach better suited to the resolution structure of our data, which allows significantly reducing computing times. Specifically, we discretized landscape variables into ecological classes and directly optimized their resistance weights using commute distance matrices and Generalized Least Squares (GLS) models with Maximum Likelihood Population Effects (MLPE).

First, we used R package *Terra* to rasterize the spatial data described above, where an integer value was attributed to each class: rivers in two classes, roads in two classes, land cover in 10 classes, ridge lines in five classes (three in Guadarrama and two in Alcarria), and slope in four classes. To build resistance surfaces, we used an optimization process with Genetic Algorithms as implemented in R package *GA*^69^, where the search parameter space considers values between 0 and 1 defining the relevance of each class to derive optimal resistance surfaces explaining genetic differentiation among populations. We applied a function that generates a transition matrix of resistance distances between geolocations of genotyped populations in the rasterized layers using R package *gdistance*^70^, by defining the average cost (*commuteDistance*) of moving from each pixel to its neighbouring eight cells/pixels. The *gdistance* algorithm uses a conductance (that is, the inverse of resistance) surface to model landscape permeability, which is used to derive resistance distances between locations through commute distances^70^. We used conductance surfaces to depict optimized landscape permeability to movement, avoiding representation issues with infinite resistance when conductance is 0 (impermeable barriers). As optimizing function for Genetic Algorithms, we used the log-likelihood of a generalized least squares regression with Maximum likelihood population effects (MLPE) using R package *corMLPE* https://github.com/nspope/corMLPE), which controls for the lack of independence of samples within a pairwise matrix^71^.

Finally, we built a data frame with genetic distances (G´_ST_ values), resistance matrices, an Euclidean distance matrix among samples (to test for IBD = Isolation By Distance, with R package *stats*), and a matrix combining all pairs of sampling populations. We assessed five non-mutually exclusive hypotheses explaining how specific landscape features shape patterns of genetic structure in *B. spinosus* in each study area. These hypotheses were implemented through resistance matrices representing rivers, roads, land use, ridge lines (topography) and slope. Each landscape feature was modelled to test its potential role as a barrier or facilitator of gene flow. We defined a set of formulas of linear models reflecting specific biological expectations and describing the relationships between genetic and geographic distances and landscape resistance values across sampling population pairs. For each formula, we adjusted a GLS model based on spatial correlation that represents the dependence between pairs of sampling populations (function: *corMLPE* in R package *corMLPE*). We assessed collinearity among predictors by calculating pairwise Pearson correlation coefficients *r*^72,73^ with R package *car*^74^. We a priori discarded all models containing two or more predictors showing correlation values |*r|*>0.7^75^. Then, we calculated metrics for model selection including Akaike’s Information Criterion corrected for small sample sizes (AICc), Delta AICc (ΔAICc), Log-Likelihood (Loglik), and AICc weights, using R package *nlme*^76^. For each model, we extracted the estimated coefficients, standard errors and p-values of each coefficient. Finally, we compared the AICc of adjusted models with the null model, which describes genetic differentiation in simulations of random movements in the absence of ecological barriers.

All experimental protocols (field procedures and tissue collection) were approved by the Museo Nacional de Ciencias Naturales, Consejo Superior de Investigaciones Científicas (CSIC) and the Community of Madrid. All the methods were carried out in accordance with relevant guidelines and regulations, including ARRIVE (www.arriveguidelines.org). Sampling permits were provided by the Community of Madrid (refs 10/142121.9/20, 10/150875.9/20, 10/020158.9/21, 10/035516.9/22, 10/184709.9/22, 10/370877.9/22).

## Results

### Genetic diversity

One microsatellite marker (*Bspi4.25*) showed significant deviations from HWP across 11 of our sampling populations, as well as a proportion of missing data (14.1%) well above the rest of markers (0.19–1.69%, Supplementary Table S2). Thus, this marker was excluded from downstream analyses, rendering the genotype dataset with the remaining 11 markers (Supplementary Table S2). These microsatellites showed high polymorphism, with six to 23 alleles per locus (Supplementary Table S2). Neither of the 21 sampling populations showed significant deviations from HWP in more than two markers (Table 2), and only two sampling populations (p9 and p17) showed significant departures from LD in one and two pairs of markers, respectively. Values of genetic diversity indices - allelic richness, observed and expected heterozygosity, and inbreeding coefficient (F_IS_)—were similar in both study areas (Table 2 and Supplementary Figure S1).

### Genetic structure and population differentiation indices

**Fig. 2:**
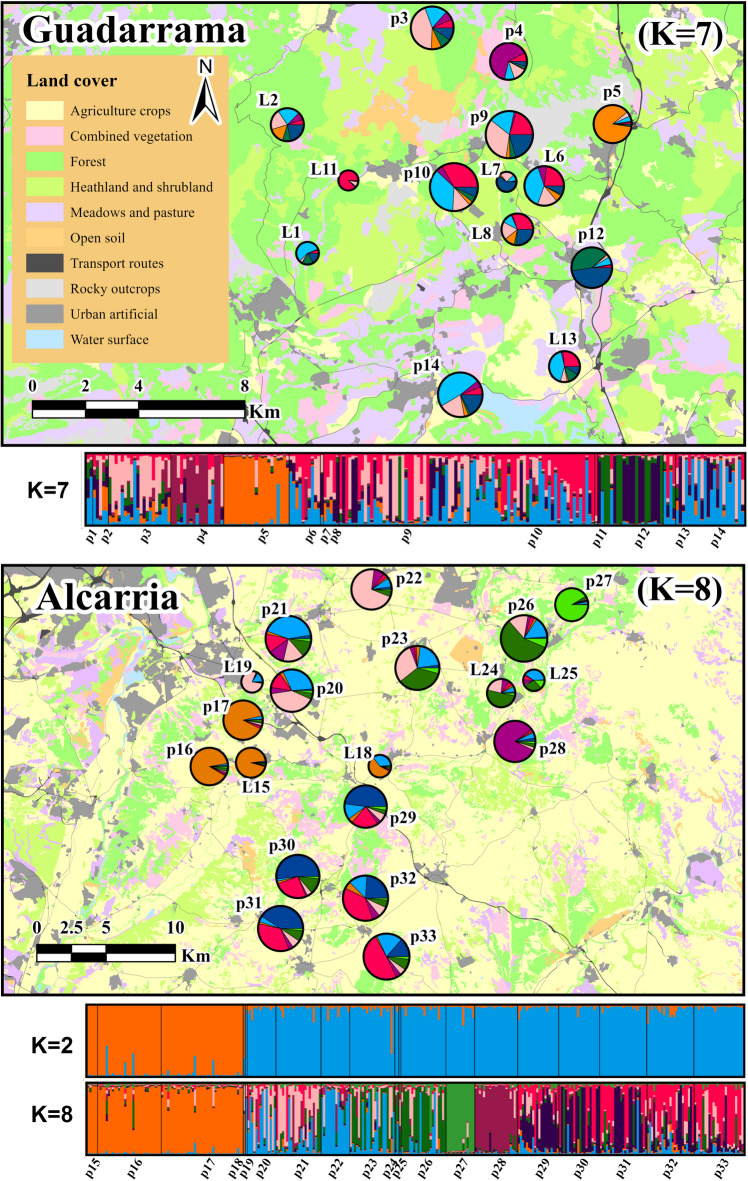
Land use/cover maps of both study areas showing sampling populations (“p”) and localities (”L”). Pie charts are coloured according to genetic ancestry proportions for K values best explaining the genetic data in each study area (see Supplementary Figure S2). The size of the pie charts is proportional to the average number of alleles per locus (Na, Table 2). Maps were built with ArcGis Pro 3.3.0.

In Guadarrama, the number of genetic clusters best explaining the data was *K* = 7 (Fig. 2a), based on both criteria (increasing likelihood and ∆K, Supplementary Fig. S2). Nevertheless, results showed no clear patterns of genetic structure across the study area, other than local differentiation of some sampling populations (such as p4, p5, or p12). In Alcarria, the best model according to the ∆K method was *K* = 2, but the highest mean estimated Ln probability of the data, L(K) corresponded to *K* = 8 (Fig. 2b; Supplementary Fig. S2). At *K* = 2, three western localities and sampling populations (L15, p16, p17) clustered together, differentiating from a second cluster formed by samples from all other sites. At *K* = 8, however, several geographically structured population groups emerged, corresponding to the west (L15, p16, p17), north (L19, p20, p21, p22), east (L24, L25, p26), and south of the study area (p29, p30, p31, p32, p33), with some localities and sampling populations displaying intermediate genetic profiles (L18, p23), or strong genetic differentiation (p27, p28) at short geographic distances (Fig. 2b).

Pairwise genetic differentiation among sampling populations was similar in the two study regions (Guadarrama: G′_ST_: 0.05–0.42, average: 0.21, D_JOST_: 0.03–0.33, average: 0.15; Alcarria: G′_ST_: 0.04–0.42, average: 0.21; D_JOST_: 0.01–0.29, average: 0.14) (Supplementary Table S4). However, average geographic distances among sampling populations in the Alcarria study region doubled those in Guadarrama (14.79 km vs. 7.03 km, Supplementary Table S5). The relationship between genetic and geographic distances (Isolation by Distance, IBD) was significant in Alcarria but not in Guadarrama (Mantel tests, Guadarrama, *R* = − 0.235, *p*-value = 0.748; Alcarria, *R* = 0.456, *p*-value = 0.001, Supplementary Fig. S3).

### Landscape genetic analyses

**Fig. 3:**
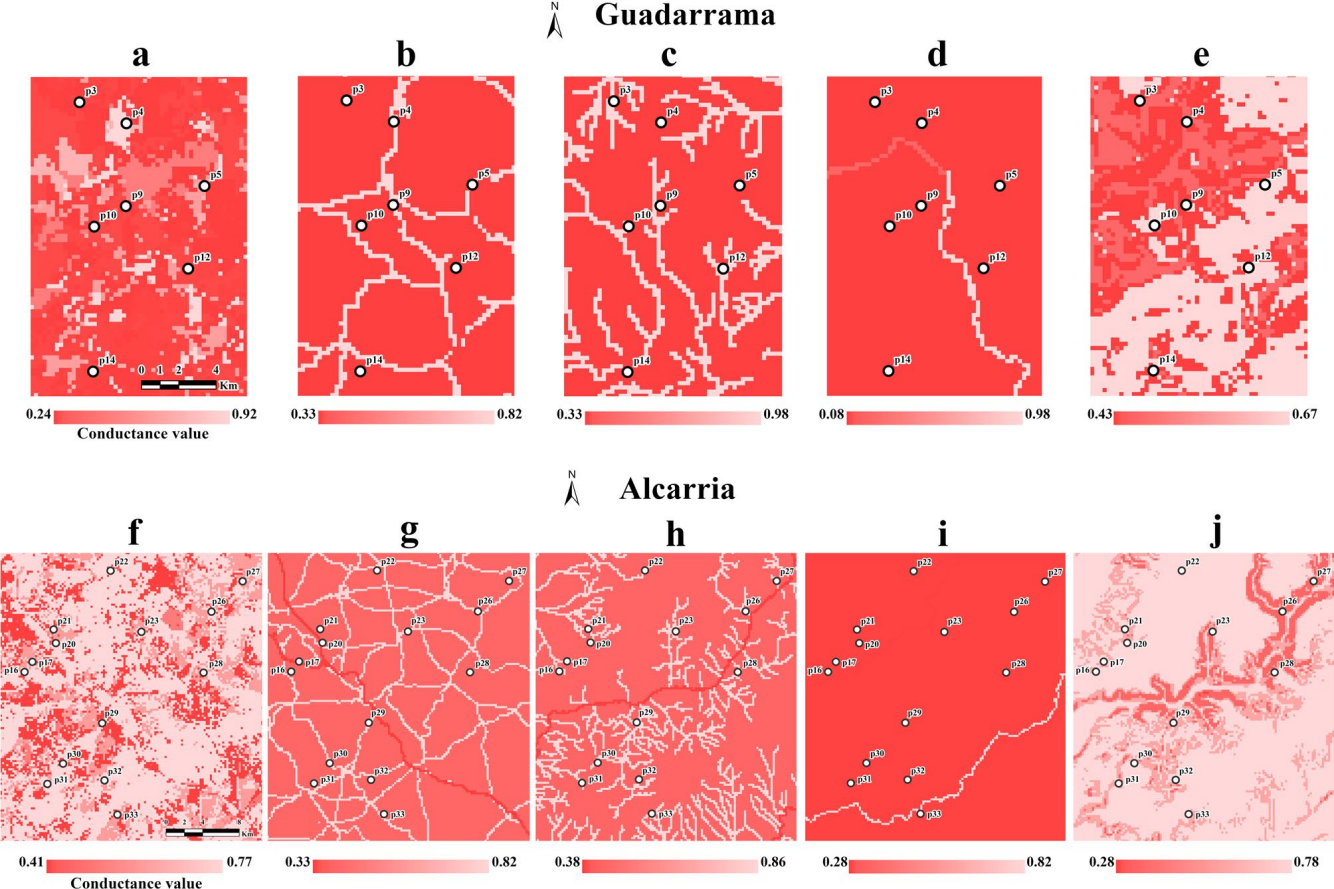
Optimized conductance maps for different landscape variables in each study area: a,f = land uses, b,g = roads, c,h = rivers, d,i = ridge lines, e,j = slopes. The colour ramp represents the degree of conductance, from low conductance (dark red) to high conductance (light red). White dots represent sampling populations (Table 2). Maps were built with ArcGis Pro 3.3.0.

The optimization of resistance surfaces revealed differences in conductance (the inverse of resistance, see Methods) across study areas (Fig. 3 and Supplementary Table S6). In Guadarrama, low conductance (values < 0.3) for populations of *B. spinosus* was associated with forests, scrublands, pastures and field crops, and moderate to high conductance with urban areas (0.66) and rocky outcrops (0.51). In turn, surfaces with mixed vegetation (complex, heterogeneous vegetation mosaics that cannot be associated with a specific land cover type, Supplementary Table S3) were associated with high conductance (0.92). In Alcarria, urban areas, combined vegetation and scrublands showed lower conductance values (around 0.41) than forests (0.63) and agriculture crops (0.77), which represent the most abundant LUC class. Highways showed the lowest conductance values in both study areas (0.33) in contrast to secondary roads, which have higher conductance (0.82). A similar pattern was observed with watercourses: larger, permanent rivers (Guadalix in Guadarrama, Tajuña in Alcarria) displayed low conductance (0.43 and 0.38, respectively), while temporary streams offered very high conductance (0.98 in Guadarrama and 0.86 in Alcarria). Regarding ridge lines, conductance across basins in Guadarrama was low in ridges with higher average elevation (conductance values = 0.08 and 0.29 for the Lozoya/Jarama and Lozoya/Guadalix ridge lines, respectively) compared to the lower-elevation ridge line (Jarama/Guadalix, conductance value = 0.92). In the Alcarria plateau, conductance in the Tajuña/Tajo ridge line (0.82) was much higher than in the Tajuña/Jarama ridge line (0.28). Finally, a progressive decrease in conductance was observed at increasing slope values. Higher conductance values were obtained in flat areas (slope: 0–10%; conductance values = 0.67 in Guadarrama and 0.78 in Alcarria), in contrast to hilly areas (slope: 30–41%; conductance values = 0.43 in Guadarrama and 0.28 in Alcarria, Fig. 3 and Supplementary Table S6).

The following predictor variables showed high pairwise correlation values and were thus not combined in the final candidate models: (1) land cover and roads, (2) land cover and slope, and (3) IBD and ridge lines in Guadarrama; and (1) IBD with most predictors (land cover, rivers, slope and ridge lines), (2) rivers and slope, (3) ridge lines and slope, and (4) ridge lines and rivers in Alcarria (Supplementary Table S7). Only the land cover model performed better that the null model in Guadarrama (Table 3). The rest of the models involving anthropogenic factors (land cover, roads), natural features (rivers, ridge lines, slope), and combined factors (including IBD) had less explanatory power than the null model. In Alcarria, all models had more explanatory power than the null model. The best models were those considering (1) land cover, (2) land cover and roads, (3) land cover and rivers, (4) IBD and roads, and (5) land cover and slope (Table 4). Coefficients, standard errors, and p-values for individual predictors in each supported model (ΔAICc ≤ 2) are provided in Supplementary Table S8.

**Table 3:** Model selection results for the IBD and IBR analyses in the Guadarrama study area, ordered by performance (from lower to higher AICc scores).

Variables	df	logLik	AICc	ΔAICc	AICc wi
**IBRlc**	**4**	**31.29**	**− 52.08**	**0**	**0.231**
**Null model (1)**	**3**	**29.42**	**− 51.42**	**0.66**	**0.166**
**IBRlc + IBRriv**	**5**	**32.37**	**− 50.73**	**1.35**	**0.118**
**IBD + IBRlc**	**5**	**32.09**	**− 50.19**	**1.89**	**0.09**
IBRroads	4	30.21	− 49.92	2.16	0.078
IBRslope	4	30.18	− 49.85	2.22	0.076
IBRriv	4	30.17	− 49.85	2.23	0.076
IBRlc + IBRridge	5	31.62	− 49.24	2.83	0.056
IBRridge	4	29.48	− 48.47	3.61	0.038
IBD	4	29.44	− 48.39	3.69	0.036
IBRroads + IBRriv	5	30.6	− 47.21	4.87	0.02
IBD + IBRroads	5	30.21	− 46.42	5.66	0.014

lc = Land uses, riv = water courses, ridge = ridge lines, df = degrees of freedom, logLik = Likelihood, ΔAICc = Delta AICc, AICc wi = AICc weight. ΔAICc scores are shown in respect to the best performing model. Models with ΔAICc < 2 are marked in bold.

**Table 4:** Model selection results for the IBD and IBR analyses in the Alcarria study area, ordered by performance (from lower to higher AICc scores).

Variables	df	logLik	AICc	ΔAICc	AICc wi
**IBRlc**	**4**	**148.53**	**− 288.60**	**0.00**	**0.275**
**IBRlc + IBRroads**	**5**	**148.92**	**− 287.14**	**1.45**	**0.133**
**IBRlc + IBRriv**	**5**	**148.82**	**− 286.93**	**1.66**	**0.120**
**IBD + IBRroads**	**5**	**148.74**	**− 286.77**	**1.83**	**0.110**
**IBRlc + IBRslope**	**5**	**148.71**	**− 286.71**	**1.88**	**0.107**
IBRroads + IBRriv	5	148.29	− 285.87	2.73	0.070
IBRlc + IBRroads + IBRslope	6	149.39	− 285.78	2.82	0.067
IBRlc + IBRroads + IBRriv	6	149.20	− 285.41	3.19	0.056
IBRriv	4	146.18	− 283.89	4.71	0.026
IBRroads	4	146.10	− 283.73	4.86	0.024
IBRslope	4	144.92	− 281.37	7.23	0.007
IBRridge	4	144.53	− 280.60	8.00	0.005
IBD	4	134.33	− 260.19	28.41	0.000
Null model (1)	3	119.93	− 233.59	55.01	0.000

lc = Land uses, riv = water courses, ridge = ridge lines, df = degrees of freedom, logLik = Likelihood, ΔAICc = Delta AICc, AICc wi = AICc weight. ΔAICc scores are shown in respect to the best performing model. Models with ΔAICc < 2 are marked in bold.

## Discussion

Landscape genetics provides insights on the role of topographical and environmental features in shaping population structure, with practical management applications. We focused on the Iberian common toad, a generalist species considered abundant in northern Spain but facing a constant decline in the central and southern two-thirds of the country, probably exacerbated by population fragmentation due to human activities and aridification, albeit the actual causes remain elusive^48,49^. We assessed functional connectivity in two areas (Guadarrama and Alcarria) with contrasting landscapes in central Iberia, representative of other regions occupied by the species in northern vs. eastern and southern Iberia, respectively. Genetic diversity indices were similar in both study regions, with no signs of inbreeding, whereas patterns of genetic structure were associated with different landscape features promoting or disrupting gene flow. Specifically, we found consistent positive effects of temporary streams and moderate slopes on gene flow, and clear barrier effects of large, permanent rivers and highways in both regions. Our results also support differential roles of land uses across study areas, as detailed below.

Among the landscape variables considered, slopes and temporary streams showed consistent positive effects on functional connectivity across both study areas. Landscape resistance was stronger at increasing slopes, as previously shown for other co-distributed amphibians^33^. In contrast, temporary streams showed high conductance values, facilitating population connectivity overall. The positive role of streams enhancing gene flow in amphibians has been previously reported^77^, especially for species breeding in lotic waters^40^, like *B. spinosus*. Indeed, well preserved temporary streams are key breeding sites for *Bufo spinosus* in central Spain^48^. While the species largely displays terrestrial activity, adequate management of stream habitats is important to increase breeding success and recruitment and to promote population connectivity, for which controlling water pollution and the spread of alien invasive species is crucial^78,79^.

We also found strong evidence of natural and artificial features acting as barriers to gene flow, especially in the Alcarria plateau. First, the Tajuña river was associated with genetic breaks in *B. spinosus*, hinting at a potential interruption of gene flow between northern (L15-p26) and southern (p28-p33) populations in this region. This permanent river is relatively deep, with fast current flow, which probably restricts adult movement across opposite shores. In addition, it hosts abundant populations of different alien invasive species (especially fish and crayfish) which prey on amphibian eggs and larvae; in fact, we have confirmed that the toads do not use the river for breeding^48,50^. This is in line with other landscape genetic studies supporting the role of large rivers in disrupting gene flow in amphibian populations^34,40,80^, even promoting lineage diversification^41,81^. Furthermore, highway A3 also seems to function as a strong barrier in the Alcarria region, with very low conductance values overall (see genetic structure results for *k* = 2 and *k* = 7). These results highlight the role of highways as barriers to gene flow in amphibians, which is largely mediated by direct mortality of high numbers of adult individuals, although amphibian casualties are often underestimated due to their small size and the short time carcasses remain in the pavement^82,83^. In this respect, the high conductance values displayed by secondary roads is surprising, because we have identified several roadkill hotspots along some of these roads in both study areas^48^. Although some anuran species can use minor roads as effective dispersal corridors in invaded landscapes – such as the cane toad in Australia^84^—this behaviour may also have high associated costs in terms of road mortality rates, as documented in *B. spinosus*^48,49^. In fact, entire populations may have become locally extinct in some areas in southern Madrid due to mass road mortalities during seasonal migrations^48,82^. Road mortality remains one of the main threats for the viability of *Bufo spinosus* populations; according to surveys in the past four decades, the species ranks in the top three vertebrates affected by this problem in Spain^82,85^. Thus, these results may represent an artifact caused by the spatial collinearity between secondary roads and suitable habitats, like some watercourses used as breeding sites by the species or secondary river valleys^39,48^, especially in sampling populations p9, p14, p23, p29 and p32. For example, in the Alcarria plateau, roads showed strong positive correlation with land-cover and river resistance surfaces (see Supplementary Table S7). Thus, the observed pattern may be driven by correlation with favourable habitat characteristics rather than by the actual use of roads as movement corridors. This could be tested with studies recording direct movements of individuals with radiotracking and disentangling the role of minor roads and other landscape features in *Bufo spinosus* dispersal.

Considering heterogeneous areas in landscape genetics studies targeting widespread species is important because it allows unravelling the relative role of landscape features compromising or promoting connectivity regardless of the spatial context vs. those specifically associated with different habitat types^8,10,11,33^. This is especially relevant when the conservation status of populations varies geographically due to the effect of local conditions and associated threats^45,46^. The abundance of *B. spinosus* in Spain decreases along north-south and west-east axes, in line with a general decline in humid conditions from the Atlantic to the Mediterranean coast^49^. In the cool, hilly landscapes of the Guadarrama mountains, the species is relatively abundant, with a largely continuous distribution^39,48^, representative of its situation in northern Iberia^86^. In contrast, populations in the Alcarria plateau and in the south and east of Madrid are more fragmented, primarily due to the loss and degradation of breeding sites^48,51^. Our results show differences in patterns of genetic structure, with higher connectivity among populations in the Guadarrama area contrasting with the marked genetic structure observed in the Alcarria plateau. This pattern may be generalizable to other regions across the species range showing similar habitat conditions and availability of breeding sites.

We found a weak influence of geography and landscape features on genetic structure in Guadarrama. In this area, the lack of IBD probably reflects high connectivity or the influence of non-spatial factors, which may explain the poor performance of IBR models. In contrast, in the Alcarria plateau, geographic and resistance distances significantly correlated with genetic distances, implying more pronounced effects of landscape features on population structure. Some of these features may act as barriers to dispersal in *B. spinosus* in the more anthropized landscapes of Alcarria, in contrast with the better connected Guadarrama populations. Although our sampling in Alcarria extended across a larger area than in Guadarrama—and concordantly showed a broader range of distances among sampling populations—, the genetic distance patterns observed in both areas illustrate that IBD was discernible even at the smallest spatial scale analysed (distances up to 14 km in Guadarrama, see Fig. S3 in Supplementary Material). Indeed, the IBD pattern remained significant after subsampling the Alcarria dataset for a spatial extent equivalent to Guadarrama, although the reduction in scale and sample size led to a decrease in the magnitude of the effect (IBD considering only sampling populations p16, p17, p20, p21, p22, p23, and p29 in Alcarria: *r* = 0.484, p-value = 0.035). Contrasting IBD patterns across study areas are consistent with findings from other comparative landscape genetic studies in amphibians, which often report differences in IBD depending on landscape heterogeneity, spatial scale and the dispersal capacities of target species^10,38,87–90^. At any rate, to rule out potential sampling effects, comparative studies should address similar sampling extents across study areas whenever possible, which requires detailed knowledge about the local distribution of target species.

Our landscape genetic methodological approach shed light on the main factors shaping functional connectivity in *B. spinosus* across contrasting landscapes. Although optimization results can be sensitive to the methodology used^91^, we opted for a simplified, standardized and reproducible framework that could be consistently applied across regions, instead of a highly parameterized model fitted for a single area. In this sense, the *ResistanceGA* framework is a complete, powerful but computationally intensive tool with potential limitations in complex scenarios, with high sensitivity to methodological decisions^92^. Our approach is very similar to *ResistanceGA*, but relies on discrete ecological classes and a direct optimization of their resistance weights using MLPE models and commute distance matrices. This simplified framework allowed us to find a balance between biological interpretability and computational efficiency, as previously shown in other landscape genetic studies of amphibians^3,10,35^.

In the Guadarrama foothills, most land cover surfaces showed high resistance to gene flow, especially those traditionally linked to silviculture (forests) and livestock (grasslands). Forests in this area are mostly monospecific and have been subdued to exploitation for centuries, lacking underbrush. In general, silvicultural practices (such as clear cuts) have been shown to be detrimental for amphibian populations^93,94^. The only land use class with high conductance in Guadarrama was combined vegetation, which occupies a small area in the study region, and could provide refuge and foraging areas in the dispersal movements of the species^95^. In contrast, in the Alcarria plateau, crop fields covering nearly two thirds of the study region showed the highest conductance scores, whereas urban surfaces, combined vegetation or grasslands showed medium-low conductance. The positive role of crop fields on connectivity is consistent with studies on other Mediterranean amphibians that co-occur with *Bufo spinosus*^10,34,35^, but contrasts with results of studies on its sister species, *Bufo bufo*, in other European regions, where crop fields are associated with high resistance to gene flow^96^. Our results illustrate the differential impacts of local land use/cover types on population structure in closely related species as well as among populations of the same species, and can be applied to design management actions promoting connectivity in areas dominated by different land use categories.

Human modification of natural habitats is producing drastic impacts on population viability in many organisms, including formerly widespread species which are increasingly affected by landscape fragmentation^33,34^. Our comparative landscape genetics study in the “not so common” toad *Bufo spinosus* underscores the need to apply general and context-dependent management actions based on knowledge of patterns of functional connectivity. Thus, preservation of key breeding sites like temporary streams and mitigation of barrier effects associated with road infrastructure are pivotal to sustain healthy, well-connected populations, whereas other measures like management of vegetation cover and the creation of artificial breeding sites may be important to prevent population isolation and local extinctions.

## Supplementary Information

Below is the link to the electronic supplementary material.

Supplementary Material 1

## Data Availability

The genotype dataset used in the present study is available in Supplementary Table S9.
