# Supplementary material for: Barriers to functional connectivity across contrasting landscapes in the widespread but declining Iberian common toad
Source: Sci Rep. 2026 Feb 19;16:7056. doi: 10.1038/s41598-026-36452-y (PMC12920771; doi:10.1038/s41598-026-36452-y)

Supplementary Information

Barriers to functional connectivity across contrasting landscapes in the widespread but declining Iberian common toad

Carlos Caballero-Díaz[1,2,3,*], Gregorio Sánchez-Montes[3], Pedro Tarroso[4,5] , Irene Castrosín[3] & Íñigo Martínez-Solano[3]

[1] Departamento de Biología, Universidad Autónoma de Madrid, C/ Francisco Tomás y Valiente, 7, 28049 Madrid, Spain

[2] Asociación Herpetológica Española (AHE). Apdo. Correos 191. 28911 Leganés, Madrid, Spain

[3] Departamento de Biodiversidad y Biología Evolutiva, Museo Nacional de Ciencias Naturales (MNCN-CSIC), c/ José Gutiérrez Abascal, 2, 28006 Madrid, Spain

[4] CIBIO, Centro de Investigação em Biodiversidade e Recursos Genéticos, InBIO Laboratório Associado, Universidade do Porto, Campus de Vairão, 4485-661 Vairão, Portugal

[5] BIOPOLIS Program in Genomics, Biodiversity and Land Planning, CIBIO, Campus de Vairão, 4485-661 Vairão, Portugal

*Corresponding author: Carlos Caballero-Díaz, carlos.caballero@uam.es, +34 91 497 6775

Scientific Reports

Supplementary Table S1. Site codes, name of localities ("L") or sampling populations ("p"), Latitude and Longitude coordinates, study area (Sa, G=Guadarrama, A= Alcarria) and number of genotyped samples (N). Site codes as in Figure 1 and Table 2.

Site	Locality/Population	Lat	Long	Sa	N
L1	Bustarviejo 1	40.83	-3.75	G	2
L2	Canencia	40.87	-3.76	G	5
p3	Garganta de los Montes	40.91	-3.69	G	20
p4	Lozoyuela	40.9	-3.66	G	17
p5	La Cabrera	40.87	-3.61	G	21
L6	Valdemanco 1	40.85	-3.64	G	10
L7	Valdemanco 2	40.85	-3.66	G	1
L8	Valdemanco 3	40.85	-3.65	G	5
p9	Valdemanco 4	40.86	-3.66	G	42
p10	Bustarviejo 2	40.85	-3.68	G	40
L11	Bustarviejo 3	40.85	-3.73	G	1
p12	Cabanillas de la Sierra	40.83	-3.62	G	21
L13	Venturada	40.79	-3.63	G	4
p14	Guadalix de la Sierra	40.78	3.68	G	22
L15	Morata de Tajuña	40.24	-3.43	A	5
p16	Arganda del Rey 1	40.26	-3.44	A	31
p17	Arganda del Rey 2	40.27	-3.43	A	40
L18	Tielmes	40.24	-3.32	A	1
L19	Arganda del Rey 3	40.28	-3.42	A	1
p20	Arganda del Rey 4	40.29	-3.4	A	14
p21	Arganda del Rey 5	40.3	-3.4	A	22
p22	Pozuelo del Rey	40.36	-3.33	A	14
p23	Valdilecha	40.3	-3.29	A	22
L24	Orusco de Tajuña 1	40.29	-3.21	A	2
L25	Orusco de Tajuña 2	40.3	-3.19	A	1
p26	Ambite 1	40.32	-3.2	A	22
p27	Ambite 2	40.35	-3.16	A	14
p28	Carabaña	40.26	-3.21	A	21
p29	Perales de Tajuña	40.21	-3.34	A	20
p30	Valdelaguna	40.17	-3.39	A	20
p31	Chinchón	40.15	-3.41	A	23
p32	Belmonte de Tajo	40.15	-3.34	A	23
p33	Villarejo de Salvanés	40.12	-3.32	A	24

Supplementary Table S2. Characterization of the 12 microsatellites used to genotype samples for this study, including multiplex reaction (Mul), locus name, fluorochrome colour (col), sequence of repeated bases (Motif), sequence of forward (F) and reverse (R) primers, number of alleles per locus (NA), range of allele sizes in base pairs, number of populations with significant deviations from Hardy-Weinberg equilibrium (HW), and proportion of missing data for each marker (% miss). Locus Bspi4.25 was excluded from downstream analyses due to widespread deviations from HW proportions across populations and a high % of missing data.

Mul	Locus	Col	Motif	Primer sequences	NA	Range	HW	% miss
1	*Bspi3.26*	6-FAM	AGT	F: 5' GAA AGT CCC ATG TCT CGT TAT GC 3'<br>R: 5' GAA AGT CCC ATG TCT CGT TAT GC 3'	9	359-386	0	1.69
1	*Bspi4.16*	NED	GATA	F: 5' AGG GCT ACA TAT CCT CTT CAG TG 3'<br>R: 5' CTA AAC TGA GAA GAT GGC AAC CC 3'	17	226-294	0	1.32
1	*Bspi4.24*	VIC	GATA	F: 5' AAA TTT GGG AGC AAT CCT GTA GG 3'<br>R: 5' GTC ACA ACT GTC CTG TTA CCT TG 3'	15	222-286	0	1.32
1	*Bspi4.28*	PET	CTAT	F: 5' GGC ATG GGT GAA TAA AGA AGT CC 3'<br>R: 5' AAA CTT AGC TCA CCT GGT CAG C 3'	16	181-237	1	1.69
1	*Bspi4.30*	6-FAM	TCTA	F: 5' CAC AGC CCT TTA CAA TCT ATC CG 3'<br>R: 5' ACA AAC AGG CAG ACA AAT ATC AG 3'	12	92-136	0	1.69
2	*Bspi3.11*	6-FAM	CTT	F: 5' TTT CCT GCC TTC TTG TAA CGT TG 3'<br>R: 5' CTC ACT GTC AGC AAT GTA TGA CC 3'	8	258-288	0	0.19
2	*Bspi4.14*	VIC	GATA	F: 5' ATG AGT CTG CTA GGA ATT GTC TC 3'<br>R: 5' CTG TAG CAA TCA TCT TCT CCT GC 3'	23	288-348	0	0.38
2	*Bspi4.25*	NED	CTAT	F: 5' ATT GTG TTG TGG ATG GAA CTA GC 3'<br>R: 5' TAG AGA GAG CTG AAA TGT TGC TG 3'	18	156-236	11	14.1
2	*Bspi4.27*	PET	GATA	F: 5' GAG ACA CGT AAT CCA GAC TTT CC 3'<br>R: 5' TTA GGA CTT GTG TGA CAT CTG AG 3'	16	228-288	0	0.38
3	*Bspi3.02*	VIC	GGT	F: 5' GGA TTA GGG CAT AGA CAA CTG AC 3'<br>R: 5' CCG TCA CAG AGA AAT CAA AGG G 3'	6	287-297	0	0.38
3	*Bspi3.19*	PET	ACT	F: 5' CCG CTA CCA TTA CAA CTA CAC AG 3'<br>R: 5' TTG TCA GAA GAA AGA GTG ATC GC 3'	8	364-409	5	0.38
3	*Bspi4.29*	VIC	TAGA	F: 5' CCC TTT CTA TGT CAC CTC TGT AC 3'<br>R: 5' ACA GTG CCA TAT CTT CAG TGT TG 3'	22	190-258	0	0.56

Supplementary Table S3. SIOSE (land uses) reclassification table.

Code	Class	Reclass
150	Asentamiento agrícola y huerta	
210	Cultivo herbáceo	
220	Invernadero	
231	Frutal cítrico	
232	Frutal no cítrico	
233	Viñedo	**Agriculture crops**
234	Olivar	
235	Otros cultivos leñosos	
236	Combinación de cultivos leñosos	
250	Combinación de cultivos	
260	Combinación de cultivos y vegetación	
351	Playa, duna o arenal	**Beach and sand**
340	Combinación de vegetación	**Combined vegetation**
311	Bosque de frondosas	
313	Bosque mixto	**Forest**
312	Bosque de coníferas	
330	Matorral	**Heathland and shrubland**
353	Desarbolado por incendios	
240	Prado	**Meadows and pasture**
320	Pastizal o herbazal	
354	Suelo desnudo	**Open soil**
161	Red Viaria o ferroviaria	**Transport routes**
352	Roquedo	**Rocky outcrops**
516	Glaciar/nieve perpetua	**Snow Ice**
111	Casco	
112	Ensanche	
113	Discontinuo	
114	Zona Verde urbana	
121	Instalación agrícola/ganadera	
122	Instalación forestal	
123	Extracción Minera	**Urban artificial**
130	Industrial	
140	Servicio dotacional	
162	Puerto	
163	Aeropuerto	
171	Infraestructura de suministro	
172	Infraestructura de residuos	
411	Zona húmeda y pantanosa	
412	Turbera	
413	Marisma	
414	Salina	
514	Lámina de agua artificial	**Water surface**
511	Curso de agua	
512	Lago o laguna	
513	Embalse	
515	Mar	

Supplementary Table S4. Pairwise genetic distances between sampling populations in both study areas. Hedrick´s G´ST and DJOST values are shown above and below the diagonal, respectively.

Site (Guadarrama)	p3	p4	p5	p9	p10	p12	p14
p3		0.2	0.17	0.09	0.12	0.2	0.08
p4	0.09		0.39	0.27	0.22	0.39	0.16
p5	0.14	0.27		0.26	0.31	0.42	0.25
p9	0.05	0.16	0.24		0.11	0.18	0.05
p10	0.09	0.11	0.23	0.03		0.28	0.1
p12	0.12	0.28	0.33	0.13	0.23		0.22
p14	0.05	0.11	0.19	0.03	0.04	0.14	

Site (Alcarria)	p16	p17	p20	p21	p22	p23	p26	p27	p28	p29	p30	p31	p32	p33
p16		0.09	0.26	0.28	0.31	0.22	0.24	0.36	0.28	0.33	0.29	0.32	0.25	0.32
p17	0.05		0.21	0.24	0.3	0.21	0.26	0.37	0.33	0.29	0.27	0.28	0.23	0.26
p20	0.17	0.1		0.06	0.12	0.09	0.14	0.33	0.24	0.16	0.17	0.17	0.13	0.14
p21	0.17	0.14	0.01		0.15	0.11	0.15	0.36	0.2	0.15	0.22	0.19	0.16	0.12
p22	0.23	0.22	0.06	0.08		0.11	0.2	0.39	0.2	0.18	0.22	0.16	0.18	0.2
p23	0.12	0.16	0.05	0.08	0.08		0.04	0.24	0.22	0.14	0.16	0.16	0.14	0.17
p26	0.14	0.19	0.12	0.08	0.15	0.03		0.24	0.22	0.18	0.17	0.19	0.15	0.21
p27	0.22	0.24	0.25	0.26	0.27	0.18	0.15		0.42	0.35	0.31	0.35	0.39	0.38
p28	0.17	0.26	0.18	0.11	0.1	0.14	0.16	0.25		0.25	0.24	0.25	0.2	0.21
p29	0.28	0.22	0.12	0.07	0.11	0.1	0.16	0.25	0.16		0.11	0.07	0.07	0.14
p30	0.21	0.2	0.09	0.13	0.15	0.11	0.13	0.16	0.18	0.06		0.05	0.1	0.12
p31	0.24	0.2	0.12	0.13	0.07	0.12	0.14	0.25	0.16	0.03	0.02		0.08	0.07
p32	0.18	0.16	0.09	0.07	0.1	0.1	0.12	0.27	0.12	0.03	0.07	0.06		0.06
p33	0.23	0.18	0.05	0.06	0.09	0.12	0.19	0.29	0.11	0.08	0.09	0.04	0.02	

Supplementary Table S5. Pairwise geographic distances (km) between sampling populations in both study areas.

Site (Guadarrama)	p3	p4	p5	p9	p10	p12	p14
p3							
p4	3.21						
p5	7.78	4.58					
p9	6.19	4.28	4.76				
p10	6.22	5.16	6.43	1.68			
p12	11	8.38	5.47	5.01	6.01		
p14	14	12.7	11.7	8.41	7.83	6.89	

Site (Alcarria)	p16	p17	p20	p21	p22	p23	p26	p27	p28	p29	p30	p31	p32	p33
p16														
p17	0.9													
p20	4.5	3.61												
p21	5.38	4.49	1.51											
p22	13.83	12.93	9.43	8.48										
p23	13.36	12.56	9.21	9.32	6.71									
p26	21.29	20.5	17.15	17.16	11.71	7.95								
p27	25.4	24.58	21.15	21	14.51	12.05	4.34							
p28	19.68	19.08	16.52	17.15	15.21	8.82	7.24	10.69						
p29	10.5	10.33	9.92	11.32	15.85	10.68	16.53	20.83	11.76					
p30	11.38	11.77	13.42	14.92	21.56	17.36	23.56	27.86	18.46	7.03				
p31	13.29	13.79	15.79	17.26	24.15	20.01	26.12	30.41	20.8	9.61	2.66			
p32	15.1	15.26	15.9	17.39	22.46	17.04	21.79	25.9	15.64	6.62	5.13	6.18		
p33	19.45	19.66	20.39	21.87	26.7	20.96	24.83	28.72	18.09	10.94	8.63	8.39	4.49	

Supplementary Table S6. Landscape variables tested in connectivity analyses, with information about the percentage of pixels occupied in each study area and the final optimized conductance value of each class.

Variable	Classes	% pixels Guadarrama	% pixels Alcarria	Conductance Guadarrama	Conductance Alcarria
**Land uses**	Agriculture crops	0.113	0.644	0.263	0.776
	Combined vegetation	0.063	0.077	0.915	0.414
	Forest	0.334	0.14	0.247	0.632
	Heathland and shrubland	0.179	0.089	0.292	0.415
	Meadows and pasture	0.149	0.023	0.279	0.638
	Open soil	0.024	0.015	0.752	0.554
	Transport routes	0.012	0.013	0.727	0.472
	Rocky outcrops	0.059	0	0.511	0.55
	Urban artificial	0.067	0.058	0.656	0.41
	Water surface	0.016	0	0.243	0.514
**Roads**	Highway	0.03	0.02	0.33	0.331
	Secondary roads	0.106	0.12	0.819	0.824
**Water courses**	Permanent rivers	0.013	0.018	0.425	0.379
	Temporary Streams	0.188	0.175	0.976	0.862
**Ridge line**	Lozoya vs Jarama river basins	0.007	Na	0.08	Na
	Lozoya vs Guadalix river basins	0.01	Na	0.286	Na
	Jarama vs Guadalix river basins	0.019	Na	0.918	Na
	Jarama vs Tajuña river basins	Na	0.007	Na	0.275
	Tajuña vs Tajo river basins	Na	0.012	Na	0.824
**Slope**	0-10 %	0.724	0.454	0.672	0.781
	10-20 %	0.188	0.282	0.434	0.613
	20-30 %	0.088	0.189	0.497	0.406
	30-41 %	0	0.075	0.433	0.28

Supplementary Table S7. Pearson pairwise correlation coefficients between predictor variables. Values for Guadarrama and Alcarria are shown above and below the diagonal, respectively.

Variable	IBD	IBR land cover	IBR roads	IBR rivers	IBR ridge lines	IBR slope
IBD	1	0.63	0.4	0.41	0.73	0.21
IBRlc	0.72	1	0.82	-0.12	0.64	0.78
IBRroads	0.49	0.68	1	-0.41	0.31	0.73
IBRriv	0.87	0.66	0.56	1	0.53	-0.24
IBRridge	0.88	0.69	0.59	0.94	1	0.31
IBRslope	0.79	0.63	0.35	0.88	0.83	1

Supplementary Table S8. Coefficients, standard errors, and p-values for individual predictors within each model in Guadarrama (up) and Alcarria (down) study areas. Models are ordered by performance (see Tables 3 and 4, respectively). Coefficients from models with ΔAICc < 2 are marked in bold.

Model (Guadarrama)	Term	Estimated coefficient	Std.Error	p-value
**IBRlc**	**(Intercept)**	**0.353**	**0.077**	**0.000**
**IBRlc**	**IBRlc**	**0.000**	**0.000**	**0.040**
**1**	**(Intercept)**	**0.212**	**0.058**	**0.002**
**IBRlc + IBRriv**	**(Intercept)**	**0.292**	**0.087**	**0.003**
**IBRlc + IBRriv**	**IBRlc**	**0.000**	**0.000**	**0.037**
**IBRlc + IBRriv**	**IBRriv**	**0.000**	**0.000**	**0.180**
**IBD + IBRlc**	**(Intercept)**	**0.366**	**0.076**	**0.000**
**IBD + IBRlc**	**IBD**	**0.000**	**0.000**	**0.246**
**IBD + IBRlc**	**IBRlc**	**0.000**	**0.000**	**0.021**
IBRroads	(Intercept)	0.289	0.081	0.002
IBRroads	IBRroads	0.000	0.000	0.222
IBRslope	(Intercept)	0.241	0.058	0.001
IBRslope	IBRslope	0.000	0.000	0.230
IBRriv	(Intercept)	0.148	0.078	0.072
IBRriv	IBRriv	0.000	0.000	0.246
IBRlc + IBRridge	(Intercept)	0.251	0.149	0.110
IBRlc + IBRridge	IBRlc	0.000	0.000	0.050
IBRlc + IBRridge	IBRridge	0.000	0.000	0.441
IBRridge	(Intercept)	0.271	0.164	0.115
IBRridge	IBRridge	0.000	0.000	0.708
IBD	(Intercept)	0.219	0.066	0.004
IBD	IBD	0.000	0.000	0.831
IBRroads + IBRriv	(Intercept)	0.224	0.113	0.064
IBRroads + IBRriv	IBRroads	0.000	0.000	0.387
IBRroads + IBRriv	IBRriv	0.000	0.000	0.416
IBD + IBRroads	(Intercept)	0.288	0.085	0.003
IBD + IBRroads	IBD	0.000	0.000	0.984
IBD + IBRroads	IBRroads	0.000	0.000	0.245

Model (Alcarria)	Term	Estimated coefficient	Std.Error	p-value
**IBRlc**	**(Intercept)**	**-0.035**	**0.036**	**0.333**
**IBRlc**	**IBRlc**	**0.000**	**0.000**	**0.000**
**IBRlc + IBRroads**	**(Intercept)**	**-0.047**	**0.037**	**0.206**
**IBRlc + IBRroads**	**IBRlc**	**0.000**	**0.000**	**0.020**
**IBRlc + IBRroads**	**IBRroads**	**0.000**	**0.000**	**0.323**
**IBRlc + IBRriv**	**(Intercept)**	**-0.024**	**0.039**	**0.542**
**IBRlc + IBRriv**	**IBRlc**	**0.000**	**0.000**	**0.024**
**IBRlc + IBRriv**	**IBRriv**	**0.000**	**0.000**	**0.408**
**IBD + IBRroads**	**(Intercept)**	**-0.216**	**0.068**	**0.002**
**IBD + IBRroads**	**IBD**	**0.000**	**0.000**	**0.001**
**IBD + IBRroads**	**IBRroads**	**0.000**	**0.000**	**0.000**
**IBRlc + IBRslope**	**(Intercept)**	**-0.044**	**0.038**	**0.245**
**IBRlc + IBRslope**	**IBRlc**	**0.000**	**0.000**	**0.007**
**IBRlc + IBRslope**	**IBRslope**	**0.000**	**0.000**	**0.509**
IBRroads + IBRriv	(Intercept)	-0.021	0.037	0.574
IBRroads + IBRriv	IBRroads	0.000	0.000	0.033
IBRroads + IBRriv	IBRriv	0.000	0.000	0.029
IBRlc + IBRroads + IBRslope	(Intercept)	-0.065	0.037	0.084
IBRlc + IBRroads + IBRslope	IBRlc	0.000	0.000	0.316
IBRlc + IBRroads + IBRslope	IBRroads	0.000	0.000	0.162
IBRlc + IBRroads + IBRslope	IBRslope	0.000	0.000	0.250
IBRlc + IBRroads + IBRriv	(Intercept)	-0.036	0.040	0.368
IBRlc + IBRroads + IBRriv	IBRlc	0.000	0.000	0.177
IBRlc + IBRroads + IBRriv	IBRroads	0.000	0.000	0.342
IBRlc + IBRroads + IBRriv	IBRriv	0.000	0.000	0.426
IBRriv	(Intercept)	0.031	0.031	0.310
IBRriv	IBRriv	0.000	0.000	0.000
IBRroads	(Intercept)	-0.044	0.038	0.247
IBRroads	IBRroads	0.000	0.000	0.000
IBRslope	(Intercept)	-0.045	0.039	0.259
IBRslope	IBRslope	0.000	0.000	0.000
IBRridge	(Intercept)	-0.053	0.040	0.192
IBRridge	IBRridge	0.000	0.000	0.000
IBD	(Intercept)	0.141	0.030	0.000
IBD	IBD	0.000	0.000	0.000
1	(Intercept)	0.209	0.029	0.000

Supplementary Table S9. Genotype matrix for 531 individuals scored at 12 microsatellites, indicating sample code and sampling population (p) / locality (L) of origin. Missing data = 0.

Sample	pop	Bspi3_02		Bspi3_11		Bspi3_19		Bspi3_26		Bspi4_14		Bspi4_16		Bspi4_24		Bspi4.25		Bspi4_27		Bspi4_28		Bspi4_29		Bspi4_30	
ccd504	L1	288	288	261	261	385	385	377	380	308	316	250	262	246	282	212	212	260	264	217	229	206	226	104	104
ccd505	L1	288	288	261	261	385	385	377	380	324	328	250	262	246	282	212	212	260	264	181	217	226	250	104	136
Bscan1	L2	288	293	261	276	385	385	359	377	328	332	246	258	242	262	0	0	256	276	209	229	210	226	104	116
Bscan2	L2	288	288	261	261	385	385	359	362	325	328	246	246	246	246	180	180	232	260	185	209	230	230	92	120
ccd1146	L2	288	288	261	276	385	385	362	377	288	316	254	258	258	270	0	0	248	268	185	213	210	230	92	120
ccd849	L2	294	294	261	261	391	391	365	377	328	328	246	254	258	270	172	172	248	260	189	213	222	226	100	116
ccd850	L2	291	294	261	276	391	391	365	377	312	328	230	258	246	262	184	188	240	248	213	232	210	210	104	104
ccd1147	p3	288	294	261	261	391	391	377	377	316	324	230	258	246	262	172	184	244	244	201	221	226	226	96	96
ccd1148	p3	288	294	261	261	385	385	359	362	312	328	250	258	254	270	172	172	248	256	181	213	206	230	96	108
ccd1149	p3	294	294	261	261	385	385	362	377	304	308	258	258	262	270	0	0	236	248	185	213	210	226	96	124
ccd1150	p3	288	294	261	261	385	385	359	377	308	308	250	258	246	270	172	188	240	264	193	213	206	230	104	120
ccd1151	p3	294	294	261	276	376	385	362	377	312	324	230	246	262	274	184	184	268	272	201	213	214	226	92	112
ccd1152	p3	288	293	261	261	385	385	377	377	316	324	230	258	242	262	172	172	244	252	201	221	206	238	96	96
ccd1153	p3	288	288	261	261	391	391	359	377	325	328	250	254	246	246	212	212	248	248	181	213	206	214	96	120
ccd1154	p3	288	288	261	261	385	385	359	380	304	304	254	258	258	270	0	0	232	260	185	213	206	238	104	124
ccd1155	p3	288	294	261	261	379	379	377	377	308	348	250	250	254	266	0	0	256	260	181	225	206	234	100	112
ccd1156	p3	288	294	261	261	385	385	0	0	317	328	0	0	0	0	0	0	256	260	0	0	218	226	0	0
ccd1157	p3	294	294	261	261	385	385	362	362	316	344	254	258	246	270	0	0	244	244	201	213	210	230	96	104
ccd1158	p3	288	294	261	261	0	0	362	380	304	304	250	250	242	270	172	172	244	248	197	213	206	226	120	124
ccd1159	p3	288	294	261	261	385	385	362	380	308	324	230	258	258	270	192	192	232	268	181	213	194	218	96	120
ccd1160	p3	288	291	261	261	376	385	359	380	324	328	254	262	270	274	192	192	248	256	209	213	206	210	112	116
ccd1161	p3	288	294	261	273	385	385	362	374	317	328	254	258	270	270	188	192	240	256	201	213	210	222	108	116
ccd1162	p3	291	291	261	261	385	385	359	377	308	312	250	266	254	274	172	172	236	264	181	213	194	226	96	132
ccd1163	p3	288	293	261	261	376	376	377	377	316	316	230	230	246	274	180	180	240	252	185	213	194	210	120	132
ccd1164	p3	288	288	261	261	391	391	377	377	328	328	250	258	246	246	188	188	248	260	181	213	206	214	104	104
ccd1166	p3	288	294	261	261	379	379	362	377	308	328	246	254	262	262	0	0	248	260	185	217	226	230	92	104
ccd1167	p3	288	294	261	261	385	385	362	377	304	312	250	258	254	270	192	192	248	256	185	213	206	222	96	124
Bsloz1	p4	288	288	261	261	385	385	359	362	304	324	258	258	242	246	0	0	232	260	185	185	194	214	112	116
Bsloz10	p4	288	288	261	261	379	379	362	365	304	328	246	258	262	270	172	172	256	268	209	213	214	226	108	116
Bsloz11	p4	288	288	261	261	379	379	362	365	304	308	246	258	262	270	172	184	256	260	185	213	214	226	92	116
Bsloz12	p4	288	288	261	261	379	379	377	380	304	324	246	250	242	270	0	0	232	268	193	213	194	194	112	120

Bsloz2	p4	288	294	261	261	379	379	362	380	308	308	250	250	258	258	0	0	240	240	193	236	226	226	104	104
Bsloz3	p4	288	288	261	261	379	379	362	377	308	324	246	258	242	270	172	172	240	260	185	213	206	226	108	116
Bsloz4	p4	288	288	261	261	385	385	365	377	308	328	246	250	258	270	0	0	240	260	185	213	198	226	92	108
Bsloz5	p4	288	288	261	261	379	379	362	377	324	328	250	258	242	270	172	172	240	260	185	213	214	226	108	116
Bsloz6	p4	288	288	261	261	379	379	359	377	304	328	250	258	246	270	172	172	232	240	185	213	194	222	104	112
Bsloz7	p4	288	288	261	261	379	379	362	365	304	328	246	258	262	270	184	184	256	260	209	213	206	226	108	116
Bsloz8	p4	288	288	261	261	379	379	362	377	324	328	250	258	262	270	172	172	256	260	209	213	206	226	108	116
Bsloz9	p4	288	288	261	261	379	379	362	365	304	328	246	258	242	270	172	184	256	268	209	213	198	206	108	116
ccd68	p4	288	288	261	273	385	385	362	374	320	344	246	258	258	258	0	0	248	252	197	201	226	230	104	120
ccd69	p4	288	294	276	282	385	385	377	377	304	328	230	246	246	258	172	188	248	264	181	193	206	210	104	104
ccd70	p4	288	288	261	282	379	379	362	365	304	304	250	258	262	270	172	172	256	260	185	213	198	214	108	116
ccd71	p4	288	288	261	261	379	379	362	365	308	324	246	258	242	270	172	172	240	268	185	213	198	214	92	116
ccd72	p4	294	294	261	261	385	385	359	362	316	324	250	258	258	262	172	184	236	256	193	197	226	230	108	120
Bscab10	p5	291	293	261	261	385	385	362	365	332	344	258	274	254	258	172	188	244	244	189	189	210	234	104	116
Bscab11	p5	293	294	261	261	364	385	377	377	304	321	258	274	254	270	172	172	244	248	209	213	222	238	116	124
Bscab12	p5	291	293	261	261	364	364	362	365	332	344	246	250	254	270	196	196	244	260	181	213	210	234	104	104
Bscab14	p5	288	293	261	261	364	385	362	377	312	328	234	258	262	270	188	196	244	248	181	213	202	222	104	116
Bscab15	p5	294	294	261	261	364	364	365	374	312	344	258	274	254	270	172	188	248	260	189	213	210	234	104	116
Bscab16	p5	291	294	261	261	364	385	377	377	304	321	258	274	262	270	172	172	244	248	209	213	202	238	116	124
Bscab17	p5	291	294	261	261	364	385	374	377	321	333	274	274	246	270	172	172	244	268	181	213	222	230	96	116
Bscab18	p5	291	293	261	261	364	385	365	374	332	344	246	274	258	270	188	188	244	244	189	213	210	234	104	104
Bscab19	p5	288	288	261	261	385	385	362	377	312	333	258	274	242	270	172	172	244	248	189	213	210	222	96	104
Bscab20	p5	288	293	261	261	385	385	362	377	321	328	258	274	254	270	172	172	244	244	209	213	222	238	116	124
Bscab22	p5	288	288	261	276	385	385	377	380	317	333	254	274	246	270	172	184	240	244	181	213	230	230	96	112
Bscab23	p5	288	293	261	261	385	385	377	377	321	328	258	274	262	270	172	172	244	248	209	213	222	238	116	116
Bscab24	p5	291	294	261	261	364	364	374	377	312	332	250	250	254	270	172	172	240	244	185	213	210	234	96	104
Bscab25	p5	291	293	261	261	364	364	365	374	317	332	246	274	258	270	172	188	248	260	189	213	210	234	104	116
Bscab26	p5	294	294	261	261	385	385	374	377	328	333	246	250	270	274	172	188	232	244	181	213	210	238	96	116
Bscab27	p5	288	294	261	276	385	385	377	377	304	333	250	250	266	270	172	196	244	264	189	213	222	230	96	116
Bscab28	p5	288	291	261	261	385	385	377	377	312	333	234	250	242	270	196	196	244	248	189	213	210	222	96	104
Bscab6	p5	294	294	261	261	379	385	377	377	321	333	286	294	222	238	184	184	244	248	0	0	0	0	0	0
Bscab7	p5	288	294	261	261	364	364	362	377	304	312	250	274	242	270	172	172	240	248	189	213	230	234	96	104
Bscab8	p5	288	291	261	261	364	385	377	377	312	328	234	258	262	270	188	196	244	248	209	213	202	238	104	116
Bscab9	p5	291	294	261	261	385	385	362	377	316	321	250	274	262	270	172	172	240	244	185	213	226	238	96	116
BB09007	L6	291	293	261	273	385	388	0	0	308	340	0	0	0	0	164	164	248	272	0	0	218	234	0	0
BB09008	L6	288	291	261	273	385	385	359	362	308	333	250	250	250	258	164	172	248	272	189	236	218	234	92	104

BB09009	L6	294	294	261	261	385	385	365	377	304	332	230	246	266	270	176	176	244	276	201	213	226	230	124	124
BB09011	L6	293	294	261	261	385	385	362	377	344	348	250	258	258	258	188	188	256	264	197	209	226	226	104	108
BB09013	L6	294	294	261	273	385	385	362	362	304	304	254	266	258	282	184	184	236	260	185	193	194	214	92	112
BB09015	L6	288	288	261	261	379	379	362	362	304	308	254	254	246	258	0	0	260	260	185	185	206	222	92	104
BB09016	L6	288	288	261	261	385	385	362	377	304	304	230	250	254	266	188	188	248	260	185	201	210	210	104	104
BB09017	L6	288	294	261	261	379	379	362	377	304	344	250	286	254	262	164	164	256	264	189	193	198	238	104	104
BB09018	L6	291	294	261	261	385	385	0	0	320	344	0	0	0	0	0	0	240	248	0	0	206	242	0	0
BB09601	L6	294	294	261	261	385	385	359	362	304	304	230	250	262	266	188	188	248	248	193	213	206	210	92	108
BB09010	L7	288	288	261	261	385	385	359	362	308	312	250	262	262	266	184	184	232	248	189	221	206	210	104	104
BB09014	L8	294	294	261	261	385	385	0	0	312	312	250	250	258	258	184	184	248	248	0	0	242	242	104	104
BB09044	L8	288	294	261	261	385	385	362	380	304	317	250	250	246	254	0	0	236	236	197	201	206	226	104	112
BB0920	L8	294	294	261	273	364	385	377	377	304	344	254	258	242	262	188	188	256	260	185	189	226	238	104	112
BB0921	L8	294	294	261	261	385	385	362	377	328	328	254	266	246	250	172	176	248	256	189	201	230	230	112	120
BB09397	L8	288	293	261	261	385	391	362	380	304	304	254	270	262	262	188	188	256	260	185	189	206	218	116	120
BB09012	p9	288	288	261	261	385	385	377	380	300	344	254	258	242	266	164	164	244	260	181	193	194	206	104	132
BB09051	p9	288	294	261	282	385	385	362	380	304	316	246	258	254	282	172	172	240	264	181	201	214	222	112	112
BB09153	p9	288	294	261	261	376	385	359	362	308	325	250	262	254	262	176	176	260	260	185	197	226	230	104	104
BB09166	p9	288	294	261	273	385	385	365	377	304	332	254	254	254	266	184	184	240	260	181	181	210	214	104	112
BB09167	p9	288	288	261	261	385	385	377	377	308	312	250	258	246	254	0	0	248	264	193	209	194	214	120	132
BB09185	p9	288	294	261	261	376	385	359	359	320	325	250	258	254	254	184	184	232	240	185	197	226	230	104	108
BB09186	p9	288	288	261	261	376	385	359	359	304	308	258	262	254	262	184	184	260	260	197	205	226	230	104	124
BB09188	p9	288	294	261	273	379	391	362	362	332	332	262	262	242	282	172	172	256	260	185	185	206	246	104	104
BB0919	p9	288	288	261	288	385	391	377	377	304	317	230	246	246	254	184	184	260	264	217	217	194	238	120	124
BB0922	p9	293	294	261	261	385	385	359	362	300	324	246	258	258	270	0	0	244	256	193	213	206	210	120	132
BB0923	p9	294	294	261	261	385	385	365	377	308	332	230	250	246	246	172	172	256	260	181	217	234	251	104	104
BB0924	p9	288	293	261	273	385	385	365	377	304	332	250	254	254	258	184	184	240	260	181	237	210	210	104	112
BB09246	p9	288	288	261	273	385	385	359	377	308	317	250	250	258	266	172	172	268	268	185	197	194	242	92	116
BB0925	p9	288	294	261	273	385	385	377	377	304	344	246	254	254	258	184	184	240	260	181	237	210	214	124	124
BB0926	p9	288	294	258	261	385	385	374	380	316	324	254	258	258	270	184	184	244	268	189	213	194	234	112	120
BB09264	p9	288	288	261	261	385	385	362	380	300	304	250	258	242	254	196	196	244	248	193	193	210	210	112	132
BB09265	p9	288	294	261	261	385	385	359	362	325	328	250	250	242	258	172	172	260	264	185	209	210	210	104	104
BB09266	p9	293	294	261	276	385	385	362	377	304	332	246	250	254	254	184	184	240	260	181	205	210	214	104	112
BB09267	p9	288	288	261	282	385	385	359	377	304	308	250	250	250	258	176	192	240	260	185	209	230	234	104	136
BB09269	p9	294	294	261	282	385	385	377	380	304	312	258	258	258	274	0	0	248	260	201	209	206	222	104	116
BB0927	p9	288	288	261	261	385	385	377	380	316	344	254	258	266	270	196	196	244	260	185	213	210	226	104	112
BB09271	p9	294	294	261	282	385	385	0	0	328	344	0	0	0	0	0	0	240	240	0	0	222	242	0	0

BB09273	p9	288	294	258	261	385	385	380	380	312	316	246	258	258	266	184	184	244	248	181	193	194	230	104	120
BB09275	p9	288	293	261	282	385	385	377	380	324	344	254	266	254	258	188	188	240	244	189	189	194	206	104	120
BB0928	p9	288	294	261	273	379	385	365	377	304	304	250	258	254	266	184	196	240	248	193	213	194	230	104	104
BB0929	p9	288	294	261	261	385	385	359	380	304	316	250	258	242	270	172	172	244	248	193	213	210	230	104	104
BB0930	p9	293	293	261	276	385	385	377	377	304	304	246	250	258	266	184	184	240	240	205	237	210	214	112	124
BB0931	p9	288	293	261	261	385	385	359	380	304	325	234	246	246	258	196	196	248	260	185	217	194	226	108	128
BB0932	p9	288	293	258	261	379	385	359	377	308	328	250	250	246	266	192	192	240	260	205	217	230	234	104	104
BB09328	p9	288	294	261	261	376	385	359	380	320	325	258	262	254	254	184	184	232	260	185	197	226	230	104	108
BB0933	p9	288	294	261	282	385	385	359	365	304	312	250	254	266	266	184	184	248	256	189	189	190	218	104	108
BB0934	p9	294	294	261	261	385	385	365	380	304	304	246	250	254	262	0	0	248	256	201	221	206	234	104	128
BB0935	p9	288	293	261	261	385	385	365	380	308	324	246	250	246	266	184	184	248	256	189	217	194	226	104	116
BB0936	p9	293	293	261	282	379	379	362	377	308	328	250	258	250	266	176	176	240	260	209	217	230	251	104	104
BB0937	p9	288	294	261	276	385	385	362	362	304	316	258	258	254	270	172	196	240	248	201	213	206	210	104	104
BB0938	p9	288	288	261	276	364	385	359	362	316	336	258	258	254	262	0	0	240	248	201	201	206	210	104	104
BB0939	p9	288	294	261	261	385	391	365	380	308	312	230	250	262	274	176	176	248	260	201	221	202	230	104	124
BB09398	p9	0	0	261	261	0	0	359	380	300	325	246	258	242	258	184	184	248	268	185	189	0	0	104	132
BB0940	p9	291	294	261	261	385	385	362	362	312	328	250	250	266	274	172	172	236	260	185	217	226	238	96	96
BB0941	p9	294	294	261	261	379	385	359	377	304	328	230	254	246	266	184	188	248	256	189	213	206	251	108	108
BB0942	p9	294	294	261	276	385	385	359	380	304	336	250	258	242	270	196	196	244	244	193	213	210	230	92	112
BB0943	p9	288	294	261	261	385	385	359	380	316	333	234	258	266	270	196	196	244	260	181	213	194	226	104	112
BB09065	p10	288	288	276	282	385	385	377	377	304	328	230	234	266	266	164	188	260	268	185	193	202	246	104	104
BB09082	p10	288	293	261	261	379	379	362	377	304	325	230	262	266	286	192	192	248	260	209	213	202	234	112	136
BB09088	p10	293	294	261	261	385	385	377	377	304	316	230	250	254	274	172	172	232	264	201	213	194	230	104	108
BB09093	p10	293	294	261	261	376	385	362	377	312	324	258	258	258	262	172	172	248	268	201	221	206	250	136	136
BB09096	p10	294	294	261	261	379	379	359	365	304	344	246	258	266	270	172	188	264	264	185	213	210	226	96	108
BB09097	p10	288	294	261	261	379	379	362	365	328	344	230	246	258	266	184	184	260	260	181	217	226	238	104	112
BB09100	p10	288	294	261	273	385	385	377	377	320	336	254	282	266	270	0	0	232	268	213	221	194	210	108	136
BB09101	p10	288	291	261	276	379	379	377	377	304	344	230	262	246	266	188	188	256	260	197	213	198	238	96	104
BB09107	p10	288	294	261	261	379	385	0	0	304	324	0	0	0	0	172	188	256	256	0	0	222	242	0	0
BB09111	p10	294	294	261	261	385	385	362	374	304	304	230	230	266	270	196	196	248	260	213	217	230	238	116	120
BB09115	p10	288	294	261	261	385	385	365	377	304	316	230	230	258	258	172	172	236	256	185	201	222	226	104	112
BB09116	p10	288	294	261	273	385	385	359	380	324	332	246	254	258	266	188	188	236	240	201	217	0	0	108	120
BB09117	p10	288	291	261	261	379	379	380	380	321	328	230	254	246	250	172	184	248	260	197	209	194	230	104	116
BB09118	p10	294	294	261	273	385	385	362	377	321	332	254	262	242	270	172	188	236	256	185	213	206	238	112	116
BB09119	p10	288	294	261	282	385	385	359	377	304	304	230	258	246	266	172	172	256	260	181	213	194	222	104	104
BB09121	p10	288	294	261	261	385	385	362	374	304	316	254	266	242	270	184	196	252	260	189	213	230	234	104	104

BB09124	p10	288	294	261	261	391	391	359	374	308	325	250	258	242	250	172	184	256	256	197	209	194	226	104	120
BB09126	p10	294	294	261	261	385	385	359	359	312	312	258	262	242	266	200	200	244	256	189	213	210	226	108	108
BB09136	p10	288	288	261	261	391	391	380	380	304	304	230	230	246	258	0	0	236	240	201	213	214	230	0	0
BB09142	p10	293	294	261	261	379	379	362	362	308	329	230	230	262	266	0	0	256	260	185	213	194	226	104	120
BB09145	p10	291	294	261	261	379	379	359	362	320	344	246	258	242	270	184	184	240	248	185	213	206	242	124	128
BB09171	p10	288	288	261	261	379	379	380	380	328	344	230	258	254	274	180	180	248	256	193	209	214	222	96	96
BB09178	p10	291	293	261	282	379	379	362	374	304	320	254	262	258	266	184	184	256	268	209	213	210	230	104	104
BB09214	p10	288	294	261	276	379	379	377	377	288	332	230	266	246	258	184	188	248	256	193	197	226	238	104	112
BB09216	p10	294	294	261	261	376	385	359	377	308	333	262	266	254	258	184	192	236	260	185	197	210	230	104	104
BB09220	p10	294	294	261	261	379	379	0	0	304	316	0	0	0	0	188	188	252	260	0	0	206	210	0	0
BB09226	p10	288	288	261	273	385	385	362	365	308	344	250	258	242	250	188	188	256	256	185	197	222	226	104	104
BB09228	p10	293	293	261	282	379	379	359	377	332	344	254	258	258	282	176	188	232	264	181	197	194	230	96	128
BB09230	p10	288	288	261	273	385	385	359	377	308	321	254	258	242	250	172	172	236	256	185	201	214	222	104	116
BB09235	p10	294	294	276	282	379	379	365	365	304	312	246	258	254	258	184	184	256	264	185	201	210	246	104	104
BB09277	p10	294	294	261	261	379	379	362	377	304	324	254	254	258	258	172	188	232	268	197	201	226	230	104	104
BB09279	p10	294	294	261	261	385	385	359	362	320	348	246	254	242	270	172	172	236	256	185	185	214	226	96	100
BB09281	p10	294	294	261	261	385	385	359	362	304	321	254	258	242	250	0	0	236	256	181	193	214	230	104	116
BB09283	p10	294	294	261	261	379	379	365	377	304	324	230	258	250	258	200	200	236	236	197	217	202	210	104	124
BB09285	p10	288	294	261	281	379	385	362	377	304	304	254	258	254	258	172	172	256	260	185	209	194	222	104	112
BB09287	p10	294	294	261	273	385	388	365	377	304	312	266	278	246	254	188	188	248	256	181	201	194	226	104	120
BB09288	p10	294	294	261	273	385	385	362	377	304	321	254	258	242	254	188	188	236	260	181	185	214	214	112	116
BB09323	p10	293	294	261	261	379	379	365	365	304	316	254	258	234	250	188	188	252	260	197	225	206	230	108	136
BB09362	p10	293	294	261	276	376	376	0	0	332	332	0	0	0	0	172	172	232	256	0	0	214	230	0	0
BB09363	p10	288	294	261	273	385	385	359	377	321	332	230	254	254	258	188	188	236	240	185	201	194	222	116	120
BB09006	L11	294	294	261	261	385	385	362	377	304	344	254	258	258	274	184	200	236	248	181	185	214	214	104	120
Bsgol1	p12	288	291	261	276	391	391	380	380	304	328	262	270	246	254	176	180	248	256	201	217	222	234	116	120
Bsgol10	p12	288	288	261	261	391	391	362	362	304	304	250	270	242	246	180	188	240	256	217	232	222	226	116	124
Bsgol11	p12	288	288	261	288	385	385	377	380	316	316	230	250	234	266	180	212	264	264	201	225	206	210	92	136
Bsgol12	p12	288	288	261	261	385	391	380	380	316	316	230	246	246	254	184	188	232	264	217	217	210	238	96	120
Bsgol13	p12	288	294	261	288	385	391	377	377	304	316	230	294	246	254	184	188	232	264	189	217	194	238	96	120
Bsgol14	p12	294	294	261	261	385	385	377	377	304	344	258	262	242	262	180	212	240	252	193	217	190	238	104	112
Bsgol15	p12	293	294	261	288	376	385	359	380	288	304	230	250	254	262	172	172	240	240	185	221	226	226	104	116
Bsgol16	p12	288	288	261	288	385	391	377	380	304	316	246	294	246	266	184	184	232	260	189	217	194	218	96	120
Bsgol17	p12	288	288	261	288	385	391	380	380	316	316	246	294	246	266	184	188	264	264	189	217	210	218	96	120
Bsgol18	p12	293	294	261	276	385	385	380	380	304	328	234	270	250	258	172	184	232	260	201	217	206	210	124	124
Bsgol2	p12	293	294	261	261	385	385	362	380	304	304	230	270	242	262	172	172	240	256	221	232	226	234	116	120

Bsgol3	p12	293	294	261	261	385	385	362	380	304	304	270	270	242	262	0	0	240	256	221	232	226	234	120	124
Bsgol4	p12	288	294	261	288	385	391	377	380	316	316	246	246	246	266	184	184	260	264	189	217	210	238	96	112
Bsgol5	p12	293	294	261	288	385	385	359	377	304	308	250	250	254	262	192	192	240	240	185	221	206	226	120	128
Bsgol6	p12	288	294	261	261	385	385	362	377	288	304	250	250	262	266	192	192	240	240	185	221	206	226	104	116
Bsgol7	p12	288	288	261	288	385	391	377	377	304	316	246	246	246	266	184	188	264	264	189	217	210	238	96	120
Bsgol8	p12	288	288	261	261	391	391	377	380	316	328	246	254	246	254	188	188	232	260	193	217	210	230	120	120
Bsgol9	p12	288	293	261	276	391	391	359	380	317	324	250	270	246	250	176	196	232	256	201	217	222	230	92	104
ccd65	p12	288	291	261	276	385	385	359	380	304	316	230	254	250	262	172	172	232	240	189	221	226	234	124	136
ccd66	p12	288	291	261	276	385	385	359	362	304	304	250	270	254	262	188	188	240	256	197	221	226	234	120	124
ccd67	p12	288	288	261	261	385	391	377	380	316	316	230	294	246	266	184	184	232	260	189	217	194	238	96	112
BsAlb1	L13	294	294	261	261	379	379	359	380	312	324	230	230	246	246	184	184	260	264	209	209	206	234	96	104
BsAlb2	L13	288	294	261	261	379	385	362	380	308	321	250	250	246	250	188	188	236	260	201	201	206	214	104	120
BsAlb3	L13	288	294	261	261	379	379	377	380	312	320	234	246	254	258	180	184	248	264	197	201	210	210	96	104
BsAlb4	L13	288	294	261	276	391	391	377	380	321	324	254	254	242	246	184	188	260	260	197	217	210	230	116	116
Bsgdx1	p14	288	294	261	261	385	385	359	365	304	308	234	262	250	262	172	172	232	256	185	221	202	234	92	112
Bsgdx10	p14	294	294	261	261	385	385	377	380	308	324	246	246	246	270	180	180	260	264	201	213	210	222	104	116
Bsgdx11	p14	288	293	261	261	385	385	362	362	308	308	246	250	262	262	0	0	232	260	185	221	226	230	104	120
Bsgdx12	p14	288	294	261	261	385	385	362	380	308	320	250	254	258	270	184	184	240	248	193	213	230	234	92	100
Bsgdx13	p14	288	288	261	282	385	385	362	377	304	309	238	262	234	270	200	200	248	248	201	225	210	230	92	100
Bsgdx14	p14	288	294	261	276	379	379	365	365	304	308	246	258	254	258	180	184	256	260	185	201	230	234	104	104
Bsgdx15	p14	288	293	261	282	379	379	365	377	320	324	250	262	246	262	192	192	228	248	197	201	210	230	100	112
Bsgdx16	p14	288	288	261	282	385	385	359	362	308	308	250	274	254	270	184	184	232	248	181	213	210	226	104	104
Bsgdx17	p14	294	294	261	261	379	379	359	377	312	329	246	282	254	266	172	176	248	260	193	213	202	222	104	108
Bsgdx18	p14	288	288	261	261	385	385	359	359	308	324	250	254	250	270	0	0	248	256	185	213	210	230	104	124
Bsgdx19	p14	288	291	261	282	379	379	362	377	304	340	238	250	234	270	184	184	232	248	201	225	214	234	92	136
Bsgdx2	p14	293	294	261	261	379	379	374	377	288	329	246	246	266	270	196	196	248	256	201	213	202	254	100	104
Bsgdx20	p14	288	294	261	261	385	385	377	380	324	340	250	250	242	250	0	0	240	260	193	197	230	234	104	136
Bsgdx21	p14	288	294	261	276	385	385	377	377	304	316	246	262	262	266	172	172	260	260	181	209	202	218	108	116
Bsgdx22	p14	288	288	261	261	379	379	359	362	308	308	258	262	254	266	172	176	240	248	185	213	206	226	100	124
Bsgdx3	p14	288	291	261	261	379	385	380	380	308	321	230	258	254	274	188	188	240	244	193	209	206	206	96	96
Bsgdx4	p14	288	294	261	276	385	385	359	377	304	316	262	282	262	266	172	188	260	260	181	209	202	230	108	116
Bsgdx5	p14	288	288	261	261	385	385	359	380	304	329	250	250	262	278	180	180	256	260	181	213	226	238	104	108
Bsgdx6	p14	288	288	261	288	379	385	362	365	304	329	234	258	258	266	172	180	232	260	181	213	198	210	96	104
Bsgdx7	p14	288	288	261	276	385	385	377	380	304	312	254	258	242	270	184	184	248	256	189	213	226	230	96	112
Bsgdx8	p14	288	288	261	261	379	385	359	377	329	340	246	250	250	266	180	180	248	260	185	213	202	210	104	136
Bsgdx9	p14	293	294	276	276	385	385	374	377	304	324	250	250	250	266	0	0	236	256	181	213	206	210	120	120

Bsmor5	L15	288	293	261	273	376	385	359	377	304	304	250	254	246	258	172	172	244	268	213	213	202	222	96	100
ccdbsmor1	L15	288	293	261	273	376	376	359	377	304	316	254	254	246	254	172	172	244	268	205	213	234	238	92	100
ccdbsmor2	L15	288	288	261	273	376	376	359	359	304	316	250	258	246	254	172	172	244	268	213	213	202	222	92	96
ccdbsmor3	L15	288	288	261	273	385	385	359	371	304	312	230	230	254	266	172	172	244	264	201	205	202	202	96	96
ccdbsmor4	L15	288	288	261	261	376	385	359	359	304	316	230	246	246	262	184	188	240	256	201	213	234	234	96	100
ccd401	p16	288	288	261	261	376	385	359	371	304	312	250	258	254	258	172	184	244	256	205	205	202	202	96	100
ccd402	p16	288	288	261	261	376	385	359	359	304	328	254	258	242	262	172	172	252	256	185	201	202	202	120	120
ccd403	p16	288	288	261	261	376	385	359	380	316	320	230	246	242	262	172	172	256	268	201	205	234	234	96	100
ccd404	p16	288	293	261	261	376	385	359	359	316	328	230	242	258	262	172	172	240	240	189	205	202	234	96	100
ccd405	p16	288	293	261	261	385	385	359	380	304	304	246	258	250	262	172	184	260	268	185	213	202	242	92	100
ccd406	p16	288	293	261	261	376	376	359	380	304	304	250	258	246	266	172	172	252	256	185	213	222	226	92	100
ccd407	p16	288	288	261	261	376	385	359	371	304	312	230	250	246	266	172	172	252	268	185	213	230	234	96	100
ccd408	p16	288	288	261	273	376	385	359	359	304	320	230	258	246	258	172	188	268	272	205	213	210	230	96	100
ccd409	p16	288	288	261	261	376	385	371	371	304	312	230	250	246	258	172	172	252	268	205	213	230	230	96	120
ccd410	p16	288	288	261	261	376	385	359	371	304	316	230	250	246	266	172	172	252	264	205	213	222	230	100	120
ccd411	p16	288	293	261	261	379	379	359	359	304	304	250	258	242	250	172	172	268	268	201	213	202	234	96	100
ccd412	p16	288	288	261	261	376	385	359	380	316	320	230	246	246	262	172	172	256	268	185	213	234	234	96	100
ccd413	p16	288	288	261	261	376	385	359	371	304	312	230	250	246	258	172	172	264	268	205	213	234	234	96	120
ccd414	p16	288	293	261	261	376	376	365	371	304	324	254	258	246	262	172	184	256	268	185	213	226	234	108	120
ccd415	p16	288	293	261	261	385	385	359	371	304	312	246	250	262	262	0	0	244	244	201	213	222	234	96	96
ccd416	p16	288	288	261	261	376	385	371	377	304	320	250	258	242	246	172	172	240	244	201	213	202	222	100	116
ccd417	p16	288	288	261	261	376	385	359	359	304	316	230	246	246	258	172	172	268	268	205	213	234	234	96	100
ccd418	p16	288	293	261	261	385	385	359	365	304	304	254	258	250	266	172	184	252	256	185	213	202	242	92	120
ccd419	p16	288	288	261	261	376	385	359	371	304	316	250	254	246	266	172	172	252	268	205	213	222	234	96	100
ccd420	p16	288	293	261	261	376	385	359	371	312	320	246	258	246	262	172	172	244	252	201	213	202	202	100	124
ccd421	p16	288	293	261	261	376	385	359	359	312	316	230	254	246	258	172	184	240	240	205	213	222	234	96	100
ccd422	p16	288	288	261	261	376	385	359	359	304	304	230	246	246	258	172	172	264	268	205	213	222	234	100	116
ccd423	p16	288	288	261	261	379	379	359	359	304	328	250	258	242	246	188	188	240	268	205	213	234	234	96	100
ccd424	p16	288	288	261	273	376	385	359	359	304	316	230	250	246	266	172	172	260	268	185	213	202	222	100	100
ccd425	p16	288	288	261	261	376	385	359	359	304	304	258	258	242	246	184	184	244	252	185	213	234	242	116	120
ccd426	p16	288	293	261	261	376	385	359	359	304	304	230	254	246	266	172	172	252	264	185	213	234	234	96	120
ccd427	p16	288	288	261	261	376	385	359	371	304	304	230	250	246	258	172	172	264	268	205	213	222	234	120	120
ccd428	p16	288	293	261	261	379	385	359	371	304	316	230	258	246	258	172	188	240	268	205	213	210	234	92	120
ccd429	p16	288	288	261	261	376	385	371	380	304	316	230	246	246	254	172	172	240	256	185	213	230	234	96	100
ccd430	p16	288	288	261	261	376	385	359	359	0	0	250	258	242	246	172	172	244	268	205	213	234	234	96	100
ccd431	p16	288	293	261	261	376	385	359	371	304	312	250	258	246	258	172	172	264	268	185	213	222	234	92	100

BSFV046	p17	288	293	261	261	376	385	359	365	296	328	230	258	242	246	184	184	240	256	205	217	202	210	96	112
BSFV047	p17	288	293	261	261	376	385	359	371	312	320	230	250	246	258	172	172	252	268	205	213	202	234	96	100
BSFV049	p17	288	293	261	261	385	385	359	359	320	328	230	258	246	262	188	188	240	256	201	217	210	234	96	100
BSFV051	p17	293	293	261	261	376	385	359	362	304	328	250	258	242	246	184	184	240	268	189	217	210	226	92	120
BSFV053	p17	288	288	261	261	376	376	359	359	312	324	230	258	242	246	172	188	252	268	201	213	210	234	96	112
BSFV054	p17	288	288	261	261	385	385	359	359	320	320	250	258	246	262	172	184	244	268	201	217	202	234	100	116
BSFV055	p17	288	293	261	261	376	376	359	362	316	324	246	258	242	246	184	184	240	256	197	213	210	234	112	112
BSFV057	p17	288	293	261	261	376	385	359	371	304	320	230	250	246	258	172	172	252	252	205	213	202	234	100	120
BSFV058	p17	288	288	261	261	385	385	359	362	304	328	250	258	242	262	172	188	252	268	185	201	234	238	92	96
BSFV059	p17	288	293	261	261	376	385	359	359	304	304	250	250	246	258	172	172	240	252	205	213	202	234	120	120
BSFV061	p17	288	293	261	261	385	385	359	362	312	328	242	258	242	258	172	184	240	268	185	185	234	238	92	96
BSFV063	p17	293	293	261	261	376	385	359	359	328	328	246	258	242	246	172	184	268	268	205	213	202	234	92	92
BSFV064	p17	288	288	261	261	385	385	359	377	304	320	238	246	242	242	184	208	240	268	185	205	234	234	100	116
BSFV066	p17	288	293	261	261	379	385	359	377	316	328	230	246	242	262	172	184	240	256	189	205	202	234	92	124
BSFV067	p17	288	293	261	261	376	385	365	371	304	320	258	258	242	246	184	184	240	268	205	213	202	202	116	120
BSFV069	p17	293	293	261	276	376	385	359	359	304	328	230	250	246	258	172	172	240	240	181	213	202	202	92	120
BSFV070	p17	288	293	261	261	385	385	359	359	312	316	242	250	258	266	172	232	268	268	181	189	202	242	112	132
BSFV071	p17	288	293	261	261	376	385	359	359	312	324	230	258	242	242	172	172	256	264	201	205	230	234	96	112
BSFV073	p17	288	288	261	261	385	385	359	371	304	320	254	258	242	242	184	184	240	268	185	201	202	230	96	116
BSFV077	p17	288	288	261	273	379	379	359	359	316	328	230	258	246	254	172	184	240	252	205	213	202	234	92	124
BSFV080	p17	288	288	261	261	376	385	371	380	304	320	246	258	242	246	184	184	240	268	201	213	202	234	100	116
BSFV088	p17	288	288	261	261	376	385	359	359	304	316	230	258	242	246	184	188	240	252	201	213	202	234	92	124
BSFV089	p17	288	288	261	261	376	385	371	377	296	304	246	250	242	246	184	208	240	240	185	213	202	234	92	112
BSFV090	p17	288	293	261	261	385	385	359	359	304	316	230	258	242	246	184	184	240	256	205	217	202	210	120	124
BSFV091	p17	288	293	261	261	376	376	359	359	316	328	246	250	242	246	172	172	240	252	201	213	210	234	92	112
BSFV093	p17	288	293	261	261	376	385	359	359	312	328	242	250	246	266	208	208	268	268	213	225	210	234	92	132
BSFV095	p17	293	293	261	261	376	385	359	359	304	304	242	246	246	262	0	0	244	256	201	213	230	230	112	120
BSFV105	p17	288	288	261	261	385	385	371	380	304	320	254	258	242	242	184	184	240	268	185	201	230	234	96	116
BSFV106	p17	288	288	261	261	376	385	359	362	316	328	246	258	242	242	172	172	240	252	197	205	210	234	92	112
BSFV112	p17	288	293	0	0	379	379	359	359	0	0	230	258	242	262	184	184	0	0	185	201	202	234	92	112
BSFV118	p17	288	293	261	261	376	385	359	359	304	304	230	238	258	258	172	172	240	252	181	205	202	234	120	120
BSFV120	p17	288	288	261	261	376	385	359	362	316	324	230	250	242	242	184	188	240	256	201	205	230	234	112	112
BSFV148	p17	288	288	261	261	376	385	359	359	324	328	250	258	242	258	184	184	240	244	201	201	202	234	92	112
BSFV153	p17	288	293	261	261	385	385	359	377	316	320	230	246	242	254	184	184	240	252	205	205	202	234	112	116
BSFV155	p17	288	293	261	261	385	385	359	359	304	316	238	250	254	254	172	172	240	252	205	213	202	234	92	112
BSFV160	p17	288	288	261	273	385	385	362	371	316	328	250	258	242	258	172	184	240	244	185	201	210	234	92	92

BSFV165	p17	288	293	261	261	385	385	359	371	304	312	230	250	258	258	172	172	252	252	181	205	202	234	96	120
BSFV168	p17	293	293	261	261	379	379	359	362	304	320	242	250	242	262	184	208	240	256	181	185	210	210	92	116
BSFV170	p17	288	293	261	261	376	385	359	359	316	324	246	250	242	242	184	188	256	268	197	205	210	234	112	112
BSFV171	p17	288	288	261	261	385	385	371	380	304	320	254	258	242	242	184	208	240	240	185	201	202	230	100	116
Bstie1	L18	288	293	261	261	376	391	359	371	296	316	250	254	242	246	172	172	244	256	205	213	202	242	100	136
ccd642	L19	288	293	261	261	385	385	359	374	308	312	250	254	254	262	176	176	268	268	189	205	198	234	132	136
bsvt1	p20	293	294	258	276	385	385	359	380	312	328	250	258	242	258	212	232	236	268	193	217	230	234	96	100
bsvt10	p20	294	294	261	261	385	385	359	365	304	316	250	250	242	262	172	196	264	268	185	205	210	242	120	124
bsvt11	p20	288	293	261	261	385	385	380	380	304	320	246	258	242	262	176	176	260	268	205	213	230	234	104	108
bsvt12	p20	288	293	258	261	385	388	359	365	328	332	254	258	246	266	180	180	256	268	201	213	218	238	112	136
bsvt13	p20	288	293	261	261	385	385	359	380	304	320	258	262	242	262	232	232	256	284	185	205	214	230	104	108
bsvt14	p20	288	294	261	276	385	385	365	380	304	328	238	254	258	270	176	212	232	268	181	217	210	254	108	120
bsvt2	p20	288	293	261	261	385	385	365	380	304	328	250	258	242	262	0	0	232	268	185	225	214	234	104	120
bsvt3	p20	288	288	261	273	385	385	365	377	312	325	250	254	258	266	0	0	260	276	201	217	234	247	120	132
bsvt4	p20	288	288	261	276	385	388	362	380	312	316	250	258	246	250	176	232	232	268	201	213	214	242	104	116
bsvt5	p20	288	293	261	261	385	385	365	380	312	312	250	258	254	262	0	0	232	232	193	205	210	234	104	132
bsvt6	p20	288	293	258	261	385	388	359	365	328	332	226	258	246	266	180	180	264	264	201	213	210	218	112	136
bsvt7	p20	288	293	261	261	385	385	359	365	300	304	250	254	250	262	172	172	232	240	205	213	210	238	108	120
bsvt8	p20	288	293	258	261	385	388	359	365	316	328	226	258	246	266	176	176	256	264	201	213	198	210	96	136
bsvt9	p20	288	293	258	261	385	385	365	380	304	304	258	258	258	270	232	232	232	268	193	217	198	210	120	120
ccd507	p21	288	293	258	261	376	385	365	377	300	328	238	250	250	262	0	0	260	268	189	221	214	254	96	124
ccd508	p21	288	288	258	258	385	391	362	368	312	316	250	250	246	254	172	172	244	268	193	213	210	239	112	132
ccd509	p21	288	293	261	261	376	391	374	377	316	328	250	258	246	262	232	232	268	268	213	213	230	238	104	136
ccd510	p21	293	293	261	261	364	385	359	359	304	328	238	258	242	242	196	196	240	256	193	193	238	258	96	136
ccd511	p21	288	293	261	261	385	388	359	365	316	320	250	254	246	270	176	176	256	256	213	229	218	238	104	132
ccd512	p21	288	294	258	273	385	385	359	362	328	328	254	258	262	266	176	176	240	268	181	197	214	254	96	108
ccd513	p21	293	293	273	276	364	385	362	362	304	328	246	250	262	262	196	196	236	240	193	205	210	210	120	136
ccd514	p21	293	293	258	261	364	364	365	374	300	320	250	250	246	262	176	176	256	272	213	225	210	230	104	116
ccd515	p21	288	288	258	261	364	376	359	380	300	304	250	258	242	242	172	176	256	260	185	185	198	198	104	112
ccd516	p21	288	291	261	261	385	391	380	380	304	312	250	258	246	250	172	176	256	260	197	213	198	222	108	112
ccd517	p21	288	288	261	261	364	385	362	362	316	320	238	254	246	250	212	212	248	256	185	225	198	242	116	136
ccd518	p21	288	297	258	261	385	385	380	380	300	316	250	258	246	262	176	176	248	256	185	205	210	242	96	104
ccd519	p21	288	293	261	261	364	385	362	380	304	312	238	250	242	250	196	196	232	260	185	189	210	214	104	120
ccd520	p21	288	294	258	261	385	385	362	380	312	324	250	254	250	258	212	212	236	256	201	217	242	255	104	108
ccd521	p21	288	293	261	261	379	385	359	365	304	312	250	250	262	266	192	200	244	272	193	201	218	238	104	108
ccd522	p21	288	294	258	261	385	391	359	377	304	316	250	254	242	246	188	200	244	260	197	213	218	234	132	132

ccd523	p21	288	293	261	276	379	385	359	359	304	312	250	254	250	266	200	200	244	256	201	205	214	214	108	108
ccd524	p21	293	293	261	276	385	385	359	365	300	316	250	250	250	262	196	196	236	272	205	225	210	230	132	136
ccd525	p21	288	288	261	261	376	376	359	359	304	328	250	258	242	242	184	196	256	256	185	185	218	239	104	108
ccd526	p21	288	288	258	276	376	385	365	380	312	320	238	250	254	262	196	196	236	268	197	213	210	210	104	132
ccd527	p21	288	288	258	261	385	391	368	377	304	304	250	250	246	250	172	172	260	268	185	213	238	238	104	112
ccd528	p21	288	293	261	261	385	385	359	362	304	328	246	258	242	246	232	232	228	232	181	185	230	238	104	104
ccd535	p22	288	288	261	273	385	385	359	362	304	308	246	250	262	266	176	188	256	260	205	205	210	230	104	136
ccd536	p22	291	293	261	261	385	391	359	365	312	312	250	258	246	246	184	196	256	264	181	221	210	238	104	120
ccd537	p22	288	288	261	261	385	385	365	377	312	312	250	250	262	262	188	200	256	264	205	209	198	210	104	116
ccd538	p22	288	288	261	261	385	385	359	365	304	312	250	250	262	266	188	188	256	264	201	205	210	234	100	104
ccd539	p22	288	294	261	261	385	385	359	377	316	324	250	254	262	262	200	200	236	264	189	205	210	214	100	116
ccd540	p22	288	288	261	261	385	385	365	380	304	312	250	258	258	262	184	184	260	276	193	205	210	234	116	120
ccd541	p22	294	294	258	273	385	385	359	362	312	320	250	254	242	262	176	192	256	260	189	205	210	234	104	116
ccd542	p22	288	293	261	261	385	385	359	368	304	312	254	258	246	266	0	0	260	260	193	229	214	239	104	116
ccd543	p22	288	293	261	273	385	385	359	365	320	325	250	250	246	262	176	200	240	268	193	205	214	230	120	120
ccd544	p22	288	291	261	261	385	385	365	377	304	312	250	258	258	262	196	196	260	276	185	205	214	222	116	120
ccd545	p22	288	293	258	276	376	385	359	359	304	312	250	258	250	262	188	188	260	260	193	205	214	254	104	104
ccd546	p22	288	288	261	273	385	385	359	362	304	308	246	250	262	266	188	188	232	256	181	205	214	238	104	104
ccd547	p22	288	288	261	273	385	385	359	374	308	324	250	250	246	262	0	0	260	264	181	205	230	230	120	120
ccd548	p22	288	293	261	273	385	385	359	359	312	316	250	258	262	266	192	192	232	260	181	205	214	234	96	104
Bsrej1	p23	288	288	261	261	385	385	359	365	312	328	246	250	246	254	0	0	256	272	197	213	218	242	120	136
Bsrej2	p23	288	288	261	261	385	385	365	377	304	328	230	258	250	262	0	0	244	276	189	205	238	242	116	116
Bsrej3	p23	288	288	258	261	379	385	362	377	304	328	250	258	246	270	196	196	252	260	189	213	230	238	104	116
Bsrej4	p23	288	294	261	261	379	385	359	359	304	304	254	254	246	266	0	0	256	268	209	221	214	214	92	104
ccd193	p23	288	288	261	261	376	391	359	365	304	316	250	258	246	270	196	232	272	276	189	217	198	214	92	136
ccd194	p23	294	294	261	261	385	385	359	377	304	304	246	250	246	266	184	200	252	268	193	221	198	230	104	120
ccd195	p23	288	288	261	261	379	385	359	362	312	328	250	254	262	266	0	0	260	264	189	205	202	214	120	136
ccd196	p23	288	288	261	261	385	385	377	377	300	320	246	258	250	262	192	196	252	252	193	205	238	242	104	104
ccd197	p23	288	293	261	261	379	385	359	365	300	304	250	250	262	270	0	0	248	256	181	205	210	234	92	100
ccd198	p23	294	294	261	261	385	385	365	377	312	320	254	258	242	262	196	196	268	280	189	205	210	230	120	124
ccd199	p23	288	293	261	261	385	385	377	377	316	316	254	258	246	266	184	200	240	252	193	221	198	230	104	124
ccd200	p23	288	293	261	261	385	385	365	380	312	325	246	254	242	254	0	0	252	272	197	197	198	218	120	124
ccd201	p23	293	293	261	261	385	385	359	365	304	304	250	258	262	270	0	0	256	272	205	225	226	230	92	92
ccd202	p23	294	294	261	276	385	385	365	377	304	320	254	258	262	270	184	184	264	268	205	209	198	210	92	116
ccd203	p23	288	288	261	261	385	385	359	362	316	316	250	258	246	270	0	0	236	260	213	213	230	242	116	124
ccd204	p23	288	293	261	261	385	385	359	377	304	316	230	250	246	266	232	232	228	268	193	213	198	238	92	100

ccd205	p23	288	294	261	261	385	385	365	377	312	316	226	246	250	262	0	0	260	268	201	205	198	234	100	104
ccd206	p23	288	288	261	273	385	385	377	377	312	312	226	250	246	258	192	192	264	268	193	221	198	234	104	120
ccd207	p23	288	294	261	261	376	376	368	377	304	316	246	250	246	258	196	220	228	252	193	193	198	202	104	104
ccd208	p23	288	288	258	261	376	385	362	377	300	312	258	258	246	254	196	196	244	248	193	213	230	238	104	112
ccd209	p23	288	293	261	261	385	385	359	359	304	316	254	254	246	270	196	196	244	256	213	213	226	238	100	100
ccd210	p23	294	294	261	273	385	385	359	377	304	316	246	250	262	270	192	192	228	268	205	209	198	214	104	104
Bsorus1	L24	288	294	261	261	385	385	362	374	304	328	230	246	254	266	172	172	256	260	185	193	238	242	116	128
Bsorus2	L24	288	288	261	261	379	385	365	365	304	316	230	254	258	262	192	232	268	280	181	205	230	238	100	108
Bsorus3	L25	288	288	258	261	385	385	359	359	308	340	246	250	246	250	168	184	248	260	193	213	238	246	96	100
Bsamb14	p26	288	288	261	261	364	385	359	380	316	320	258	258	246	258	172	196	268	268	181	221	214	238	104	124
ccd257	p26	288	288	261	261	379	385	368	377	288	320	246	250	266	266	180	180	244	268	189	193	214	238	96	120
ccd258	p26	288	288	261	261	379	385	365	368	304	316	230	246	246	266	0	0	240	272	213	217	226	242	116	116
ccd259	p26	288	294	258	261	376	385	359	368	304	320	246	258	246	266	168	184	228	268	201	221	230	238	104	116
ccd260	p26	288	293	261	276	385	385	362	368	312	316	250	254	246	266	0	0	272	276	185	213	214	230	116	120
ccd261	p26	288	288	258	261	385	385	359	359	316	328	254	258	254	262	176	176	248	260	189	209	230	242	100	132
ccd262	p26	294	294	261	261	385	385	365	380	288	308	246	258	246	270	176	208	256	268	185	213	218	238	96	116
ccd263	p26	293	294	261	261	385	385	365	377	300	304	230	246	262	270	188	196	244	276	181	209	230	238	104	124
ccd264	p26	288	288	261	261	379	385	365	377	304	304	230	258	246	246	184	184	228	256	217	221	210	238	104	108
ccd265	p26	288	288	261	273	379	385	377	377	304	316	250	258	262	266	180	188	260	264	193	205	210	214	104	116
ccd266	p26	288	288	261	261	379	385	365	374	308	337	230	250	246	266	192	192	228	256	213	213	214	238	96	100
ccd267	p26	288	288	261	273	385	385	362	377	304	320	230	258	246	266	172	196	256	268	213	213	238	238	104	116
ccd268	p26	288	288	261	261	379	385	368	377	288	304	246	274	246	250	184	184	232	236	189	221	230	242	104	120
ccd269	p26	288	293	261	261	385	385	365	365	324	337	230	254	246	266	176	184	228	228	181	213	210	238	96	136
ccd270	p26	288	294	261	261	379	385	359	374	304	320	246	250	246	254	176	176	232	248	193	213	210	230	96	96
ccd271	p26	288	294	261	261	385	385	377	380	304	308	246	250	246	262	172	172	232	260	189	221	210	258	136	136
ccd272	p26	288	288	261	261	385	385	362	365	312	316	230	258	258	262	192	192	256	276	189	205	210	210	104	120
ccd273	p26	288	294	261	261	376	385	359	377	300	332	226	254	242	246	184	184	272	288	197	213	210	238	128	136
ccd274	p26	288	294	261	273	379	385	359	377	304	316	246	246	246	266	188	232	248	276	181	213	202	234	104	112
ccd275	p26	288	293	261	261	379	385	377	377	304	308	246	246	246	246	188	188	232	244	181	221	238	242	116	132
ccd276	p26	288	288	261	261	385	385	374	380	300	328	258	274	246	266	180	196	272	276	213	213	238	242	100	116
ccd277	p26	288	293	261	261	385	385	377	377	304	304	246	258	242	246	188	188	228	256	217	221	210	238	104	108
Bsamb1	p27	294	294	261	261	385	385	359	359	304	328	246	258	246	250	192	192	248	248	189	213	230	230	92	108
Bsamb10	p27	294	294	261	261	385	385	365	365	320	320	246	274	246	266	0	0	248	268	181	213	230	230	108	116
Bsamb11	p27	294	294	261	261	385	385	359	365	304	328	246	250	246	266	0	0	248	248	213	213	226	230	96	108
Bsamb12	p27	291	293	261	261	385	385	359	377	304	304	250	274	246	250	188	188	256	260	197	213	230	238	92	136
Bsamb13	p27	291	293	261	261	379	385	359	365	312	328	246	250	246	246	172	172	248	256	189	213	230	230	108	136

Bsamb2	p27	288	293	261	261	385	385	365	377	304	328	246	258	246	250	192	232	248	260	189	213	226	230	92	108
Bsamb3	p27	288	294	261	261	385	385	359	359	304	328	246	274	246	250	172	172	248	256	189	213	230	230	108	116
Bsamb4	p27	294	294	261	261	385	385	359	359	304	328	258	274	246	266	192	192	248	260	181	213	226	230	92	108
Bsamb5	p27	288	288	261	261	385	391	359	365	304	320	246	250	246	266	192	232	240	248	213	213	230	238	96	120
Bsamb7	p27	291	293	261	261	385	385	359	377	304	304	258	274	246	254	172	188	256	260	197	213	230	238	92	136
Bsamb8	p27	288	288	261	261	385	385	365	374	304	316	250	274	246	254	204	204	236	268	189	213	230	230	96	124
Bsamb9	p27	294	294	261	261	385	385	359	365	304	320	246	250	246	266	184	232	248	268	189	213	230	230	96	116
ccd255	p27	294	294	261	261	379	385	359	377	304	304	246	250	246	250	172	192	240	248	189	213	230	230	116	136
ccd256	p27	291	294	261	261	379	385	359	359	304	312	246	250	246	254	188	192	240	260	197	213	230	230	92	92
Bscas1	p28	288	294	261	261	364	385	359	380	304	304	250	258	246	262	184	184	256	260	201	229	210	242	120	128
ccd235	p28	288	288	258	261	364	385	359	359	304	304	250	258	262	266	0	0	256	276	185	185	214	230	120	128
ccd236	p28	288	288	258	261	376	385	359	377	300	304	250	250	246	250	188	188	232	260	205	229	230	230	108	120
ccd237	p28	288	288	258	261	376	385	359	359	304	304	250	250	262	266	212	212	232	256	185	185	214	214	120	128
ccd238	p28	288	288	261	288	376	385	359	362	304	328	246	254	246	246	156	212	248	256	189	229	214	234	128	132
ccd239	p28	288	288	261	261	376	385	359	362	296	304	250	254	242	262	212	212	256	256	181	209	214	222	120	128
ccd240	p28	288	288	261	288	376	385	359	362	296	308	250	254	246	258	212	212	256	256	209	229	214	222	104	116
ccd241	p28	288	294	261	261	385	385	380	380	304	304	246	258	262	262	184	184	256	260	181	201	210	242	128	128
ccd242	p28	288	288	261	288	385	385	359	377	300	304	254	254	242	246	184	212	256	264	185	229	214	246	124	128
ccd243	p28	288	288	261	261	376	385	380	380	304	324	246	250	266	270	192	212	260	276	181	185	202	214	120	120
ccd244	p28	288	294	261	264	385	385	380	380	304	304	258	258	246	262	184	188	260	276	185	185	210	214	120	128
ccd245	p28	288	288	261	288	376	385	359	377	304	304	250	270	238	262	184	188	236	264	181	185	214	246	128	128
ccd246	p28	288	288	261	288	364	385	359	380	304	304	250	254	246	266	188	188	232	236	185	229	214	214	120	128
ccd247	p28	288	288	261	288	364	376	359	380	304	308	250	254	266	266	188	188	236	256	185	185	214	230	104	112
ccd248	p28	288	288	261	288	376	385	359	377	304	308	246	254	246	270	176	188	256	260	181	229	202	214	116	128
ccd249	p28	288	288	258	261	385	385	362	380	296	304	246	254	246	258	188	188	232	256	181	229	230	234	116	120
ccd250	p28	288	288	261	288	385	385	359	359	304	308	250	254	246	262	188	212	236	256	181	229	214	214	104	128
ccd251	p28	288	288	261	261	385	385	359	362	304	312	246	250	246	250	156	192	228	256	189	229	210	234	92	128
ccd252	p28	288	288	261	261	364	385	380	380	300	304	246	250	262	266	192	192	268	276	185	205	214	242	112	116
ccd253	p28	288	293	258	288	385	385	359	377	308	328	250	254	246	250	156	192	228	248	193	229	226	234	116	132
ccd254	p28	288	288	258	261	382	385	377	380	304	312	246	250	246	246	188	188	232	260	181	229	214	230	108	120
ccd119	p29	288	294	261	261	385	385	359	362	296	300	230	250	242	266	188	192	256	272	201	213	234	242	96	100
ccd120	p29	288	288	261	261	385	385	362	377	300	316	250	258	242	262	184	188	232	272	181	205	234	242	92	96
ccd121	p29	293	294	258	276	385	385	359	377	304	316	250	254	250	266	0	0	252	264	181	181	214	230	92	92
ccd122	p29	288	294	276	288	385	385	365	377	304	304	238	254	262	262	188	188	232	264	181	201	214	222	120	124
ccd123	p29	288	294	261	261	385	385	359	362	316	316	234	258	242	270	180	188	256	256	185	189	218	222	92	100
ccd124	p29	288	288	261	261	385	385	380	380	304	312	246	250	246	262	172	172	256	268	181	213	218	218	96	124

ccd125	p29	288	288	261	273	376	385	362	377	296	316	254	258	250	266	0	0	256	272	181	213	218	222	92	96
ccd127	p29	291	291	261	261	385	385	362	380	312	316	250	250	254	262	0	0	232	256	189	201	210	238	116	116
ccd128	p29	288	288	261	276	364	376	359	380	296	328	238	254	258	262	196	196	232	256	181	181	210	238	100	120
ccd129	p29	288	288	261	261	385	385	362	377	312	324	250	254	242	250	0	0	256	260	181	181	222	222	104	116
ccd130	p29	288	293	261	261	385	385	377	380	296	316	234	250	242	254	184	236	272	272	189	189	218	230	96	96
ccd131	p29	288	294	261	261	364	385	362	365	296	296	238	258	254	262	0	0	232	260	181	201	210	238	92	116
ccd132	p29	288	294	261	261	385	385	368	380	304	312	250	258	262	266	0	0	260	260	201	213	218	226	104	104
ccd133	p29	288	294	261	261	385	385	359	362	296	312	250	250	254	262	172	184	228	268	181	181	210	218	92	116
ccd134	p29	288	294	261	261	376	385	362	371	296	304	250	254	250	254	0	0	232	260	181	201	222	226	92	124
ccd135	p29	288	294	261	276	364	385	362	380	296	328	250	250	250	270	188	188	260	268	181	213	218	242	92	104
ccd136	p29	293	294	261	261	385	391	359	368	312	312	246	262	262	262	180	184	260	260	201	213	218	230	92	120
ccd137	p29	288	294	261	288	385	385	0	0	296	328	250	258	242	254	196	196	0	0	181	213	214	242	96	108
ccd138	p29	288	293	261	261	385	385	368	377	296	296	246	250	250	262	172	172	260	260	181	201	226	230	92	116
ccd139	p29	288	293	261	288	364	385	359	368	324	328	250	258	242	262	184	196	252	272	181	213	218	230	96	108
bsvalv1	p30	288	294	261	261	376	385	365	380	301	304	238	258	242	258	0	0	232	272	181	221	210	210	92	120
bsvalv10	p30	288	291	261	261	385	409	359	359	300	312	246	266	254	262	176	180	256	260	181	201	226	242	120	124
bsvalv11	p30	288	288	261	261	385	385	359	368	304	316	246	246	242	250	176	176	228	268	181	213	238	242	108	116
bsvalv12	p30	288	294	261	261	385	385	359	380	324	328	234	234	266	274	188	188	232	248	181	181	214	218	104	108
bsvalv13	p30	288	288	261	273	385	385	359	365	300	301	250	258	250	262	172	176	244	272	181	193	210	234	120	120
bsvalv14	p30	288	291	261	261	385	385	359	359	304	304	246	250	262	266	172	172	256	272	213	217	218	242	92	120
bsvalv15	p30	288	291	261	261	364	385	359	380	324	328	258	258	270	270	172	172	244	248	181	181	218	238	104	108
bsvalv16	p30	288	294	261	261	379	385	359	362	312	328	234	246	250	258	196	196	232	256	181	217	230	242	96	120
bsvalv17	p30	291	294	261	261	376	385	380	380	324	328	234	258	270	270	0	0	232	244	181	181	214	218	104	108
bsvalv18	p30	288	288	261	261	385	385	359	359	301	304	230	246	262	266	172	172	256	260	213	217	202	218	92	120
bsvalv19	p30	288	288	261	261	385	385	362	377	304	316	246	254	250	270	236	236	228	268	181	213	202	238	108	116
bsvalv2	p30	294	294	261	261	385	409	365	377	301	304	234	258	262	270	172	192	248	272	181	201	202	218	92	120
bsvalv20	p30	288	291	261	288	385	385	359	359	304	304	234	258	266	274	0	0	248	260	181	181	218	218	92	108
bsvalv3	p30	291	291	261	261	376	385	359	380	304	312	238	250	258	262	176	176	244	268	181	221	210	254	108	124
bsvalv4	p30	288	294	261	261	385	385	359	362	301	304	238	246	246	266	176	176	244	244	181	221	210	214	92	108
bsvalv5	p30	288	293	261	261	385	409	362	377	304	324	250	258	250	262	180	180	232	260	181	201	214	218	92	104
bsvalv6	p30	288	288	261	273	385	385	359	380	300	301	250	258	234	246	176	176	232	248	181	221	210	242	108	120
bsvalv7	p30	288	294	261	261	376	385	359	365	312	328	238	258	258	262	176	236	268	272	205	221	210	210	100	124
bsvalv8	p30	291	291	261	261	385	409	359	359	304	304	238	250	262	266	172	172	272	272	201	217	222	238	92	92
bsvalv9	p30	288	291	261	288	385	409	359	359	301	304	246	258	262	266	0	0	260	260	181	201	218	218	108	120
Bsvdz10	p31	291	293	258	258	385	385	365	380	308	328	230	234	266	266	180	180	256	272	181	181	210	226	96	120
Bsvdz4	p31	288	288	261	261	376	385	359	359	300	328	258	258	242	262	0	0	228	260	201	217	214	226	100	112

Bsvdz5	p31	294	294	261	261	385	385	359	368	312	328	230	246	250	258	0	0	256	256	181	221	242	242	104	108
Bsvdz6	p31	291	293	261	261	376	385	359	377	312	324	230	238	258	262	180	180	260	276	213	221	210	230	108	116
Bsvdz7	p31	288	291	261	288	385	385	359	377	301	308	230	258	242	266	236	236	252	260	213	217	210	230	120	124
Bsvdz8	p31	288	291	258	261	385	385	380	380	308	328	230	246	242	250	172	172	248	260	181	217	226	238	96	120
Bsvdz9	p31	288	288	258	261	376	385	365	365	296	308	246	250	262	262	176	176	248	268	185	201	214	230	96	120
ccd141	p31	291	294	261	261	385	385	359	380	300	301	246	266	242	254	172	172	260	260	213	217	214	222	92	124
ccd142	p31	288	291	261	288	385	385	359	377	301	316	246	266	254	270	180	180	260	260	181	213	214	222	124	124
ccd143	p31	294	294	261	261	385	409	359	377	296	312	250	254	262	262	0	0	244	260	201	213	222	242	92	104
ccd144	p31	288	288	261	261	385	385	377	380	300	312	250	258	266	266	176	180	244	260	181	181	218	242	116	128
ccd145	p31	294	294	261	261	376	385	359	377	296	300	250	250	262	262	180	180	240	260	193	201	210	226	92	92
ccd146	p31	291	291	261	261	376	385	359	368	328	328	238	250	250	266	172	180	260	272	181	217	210	238	100	100
ccd147	p31	288	288	258	261	376	385	359	380	312	324	238	258	250	262	172	172	228	256	193	201	210	214	100	104
ccd148	p31	288	291	261	261	376	385	359	377	304	324	250	258	250	262	180	180	244	256	181	181	218	226	104	120
ccd149	p31	288	288	261	288	376	409	359	377	300	301	234	258	262	274	0	0	260	260	201	205	214	226	124	124
ccd150	p31	288	293	261	261	385	385	362	365	304	308	230	246	258	262	180	192	256	260	181	181	202	238	108	120
ccd151	p31	288	291	261	273	376	385	359	359	296	301	250	266	262	270	180	180	232	260	181	201	230	234	92	124
ccd152	p31	288	288	261	261	385	385	359	359	312	324	254	258	266	266	180	180	232	264	205	213	230	234	104	104
ccd153	p31	294	294	261	273	376	409	359	359	301	312	234	254	250	262	172	172	260	272	181	201	214	222	120	128
ccd154	p31	288	291	258	261	385	385	365	380	301	324	230	246	250	266	172	172	256	260	181	181	226	238	116	120
ccd155	p31	291	294	261	261	376	385	362	362	308	312	230	250	242	250	172	172	232	264	181	217	210	242	108	116
ccd156	p31	288	288	261	261	385	385	359	362	300	312	234	266	262	262	236	236	232	256	189	197	222	238	100	124
Bsbel12	p32	288	288	258	261	376	385	359	371	304	304	234	254	242	262	192	192	256	268	205	217	214	238	96	108
Bsbel13	p32	288	294	261	276	385	385	359	365	312	324	254	258	246	262	176	176	232	276	181	217	218	230	116	116
Bsbel5	p32	288	291	261	261	376	409	362	374	300	316	246	258	242	262	172	236	256	268	201	201	214	238	112	112
ccd172	p32	288	291	261	261	379	409	359	377	300	312	234	246	246	262	172	172	260	260	181	201	230	242	108	128
ccd174	p32	288	288	261	261	385	385	359	359	324	332	246	254	242	246	196	196	228	272	213	217	210	218	104	108
ccd175	p32	288	288	261	261	385	409	359	365	304	312	230	258	242	262	188	188	232	256	193	201	214	214	96	112
ccd176	p32	288	288	261	261	376	385	380	386	296	300	254	258	258	262	184	184	256	256	181	201	210	234	92	96
ccd177	p32	288	294	261	276	385	385	362	365	316	316	250	254	246	262	0	0	232	260	181	217	214	218	100	100
ccd178	p32	288	288	261	273	385	385	365	368	300	304	230	230	242	246	196	196	256	260	205	217	202	254	120	128
ccd179	p32	288	294	258	276	385	409	365	377	324	324	230	250	262	262	172	172	244	272	181	201	210	254	92	104
ccd180	p32	288	294	258	261	385	385	359	362	316	324	254	258	246	262	176	176	232	260	181	217	218	230	100	100
ccd181	p32	288	288	261	261	385	385	359	380	312	328	254	254	262	266	172	172	256	268	201	209	202	230	100	116
ccd182	p32	288	288	261	261	385	385	359	362	304	324	234	246	242	266	192	192	232	268	213	217	210	234	96	108
ccd183	p32	288	288	261	261	376	376	380	386	296	317	230	254	242	262	0	0	256	260	201	201	230	234	96	108
ccd184	p32	288	288	261	261	376	385	359	362	296	304	250	254	246	254	172	184	232	272	205	217	218	222	112	116

ccd185	p32	288	288	261	276	385	385	359	368	312	324	230	250	254	266	0	0	260	268	181	201	214	218	92	104
ccd186	p32	294	294	261	288	385	409	359	377	301	324	234	238	262	270	172	172	232	264	181	201	218	222	92	104
ccd187	p32	288	288	261	288	385	385	362	362	324	328	250	250	238	266	236	236	232	256	205	213	202	230	92	108
ccd188	p32	288	291	261	261	385	391	359	380	300	332	258	258	246	262	184	184	232	268	189	217	202	222	100	108
ccd189	p32	288	294	258	261	385	385	359	365	300	316	246	258	246	262	176	176	260	268	201	217	210	238	100	120
ccd190	p32	288	288	258	261	385	385	368	377	304	316	250	258	242	246	184	188	256	260	181	193	218	218	92	92
ccd191	p32	288	291	261	288	409	409	359	362	304	304	234	234	258	262	0	0	256	264	201	201	218	218	92	92
ccd192	p32	288	294	258	288	376	409	368	374	296	312	238	258	258	262	196	196	252	268	181	201	230	254	96	96
bsbel1	p33	288	288	258	261	385	409	368	380	301	328	250	250	262	266	176	192	264	268	181	201	210	218	92	120
Bsbel10	p33	288	294	258	261	376	385	359	377	324	328	250	254	234	246	180	180	256	260	217	217	198	210	104	108
Bsbel11	p33	288	288	261	261	385	385	359	377	312	312	254	258	242	242	180	196	268	272	181	193	210	210	100	124
bsbel2	p33	288	291	258	261	376	385	359	362	332	332	250	258	246	258	180	180	232	272	201	217	214	214	108	108
Bsbel3	p33	288	288	261	261	385	385	359	377	304	320	246	258	242	250	188	188	232	268	213	221	214	230	108	120
Bsbel4	p33	287	287	258	276	379	385	377	386	308	328	250	254	246	246	0	0	260	260	197	221	214	238	104	124
Bsbel6	p33	0	0	258	258	376	385	359	362	304	328	226	254	246	274	188	188	256	260	189	225	226	230	108	124
Bsbel7	p33	288	291	258	261	376	409	368	377	296	328	258	258	262	262	172	188	232	248	201	201	210	242	92	108
Bsbel8	p33	288	288	261	261	376	409	359	362	308	328	250	258	234	262	168	216	256	260	201	201	210	214	116	124
Bsbel9	p33	288	288	258	261	376	385	362	377	320	320	254	254	250	258	180	180	256	260	181	221	198	234	108	124
ccd158	p33	288	293	258	261	376	385	362	362	316	328	250	254	242	262	192	196	256	264	189	201	214	222	100	116
ccd159	p33	291	294	261	261	364	376	365	377	317	324	250	250	246	266	180	180	256	272	213	217	214	218	108	120
ccd160	p33	288	288	261	261	385	409	359	362	301	312	254	258	242	262	0	0	260	272	201	201	210	214	100	120
ccd161	p33	291	291	261	261	376	385	359	368	328	332	234	238	262	266	176	176	232	256	201	205	214	214	108	132
ccd162	p33	288	294	261	261	376	385	359	359	300	312	254	254	242	266	172	172	260	268	181	193	210	214	124	128
ccd163	p33	288	291	261	261	385	385	359	359	312	328	226	254	254	274	168	180	260	264	181	213	214	226	92	124
ccd164	p33	288	288	258	261	385	385	359	380	301	328	250	250	242	266	176	176	264	272	181	193	210	230	108	108
ccd165	p33	288	291	261	261	385	385	359	362	312	312	246	258	242	258	192	196	268	272	181	193	210	214	100	124
ccd166	p33	294	294	258	261	385	385	359	380	328	332	234	250	242	242	176	176	264	268	181	193	210	230	92	108
ccd167	p33	288	291	261	261	385	385	362	386	316	332	250	254	242	258	176	192	232	260	181	201	218	242	108	124
ccd168	p33	288	288	258	261	385	385	359	377	304	324	246	250	242	246	172	188	248	260	193	217	198	226	104	108
ccd169	p33	291	291	258	261	376	385	359	362	300	328	246	254	242	262	176	176	268	268	181	201	230	242	120	132
ccd170	p33	288	288	258	261	385	385	359	359	301	317	238	254	262	262	184	184	240	248	189	201	218	230	108	120
ccd171	p33	288	288	258	261	376	385	359	386	304	304	254	258	246	274	0	0	236	256	213	225	210	230	108	112

Supplementary Figure S1. Comparison of average genetic diversity indices in sampling populations (>10 samples) of the Guadarrama and Alcarria study areas. Na = allelic richness, Ho = observed heterozygosity, He = expected heterozygosity, F_{IS} = inbreeding coefficient.

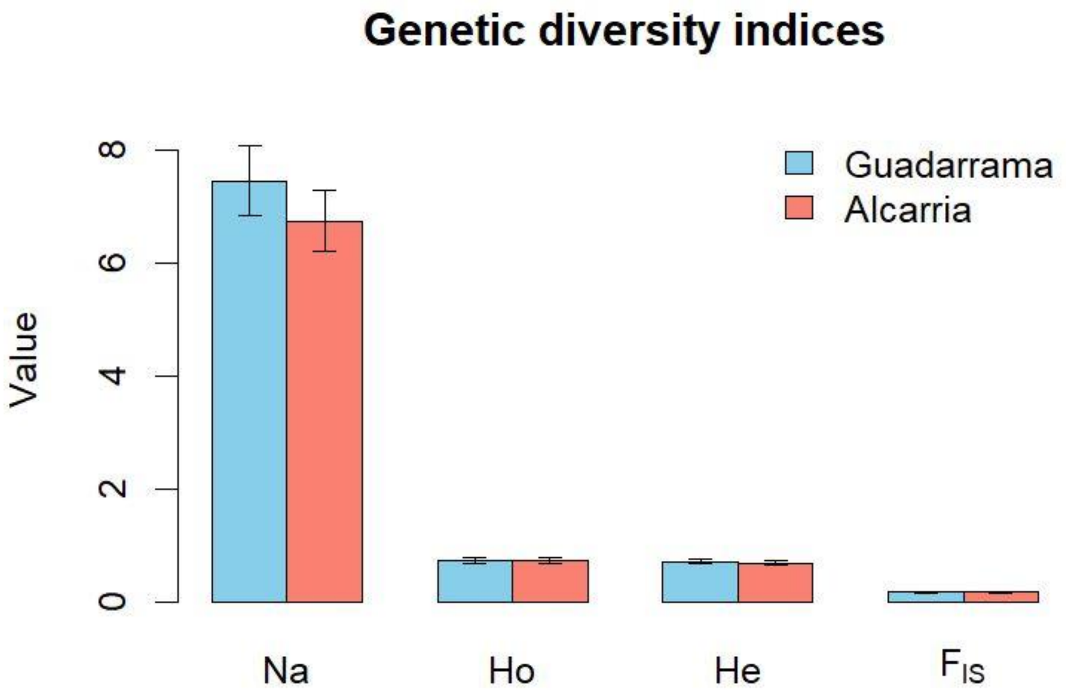

Supplementary Figure S2. Structure results. Delta K plot (A, B), mean of estimated Ln Probability of Data, L(K), plot (C, D), and summary scores (E, F) across runs in both study areas.

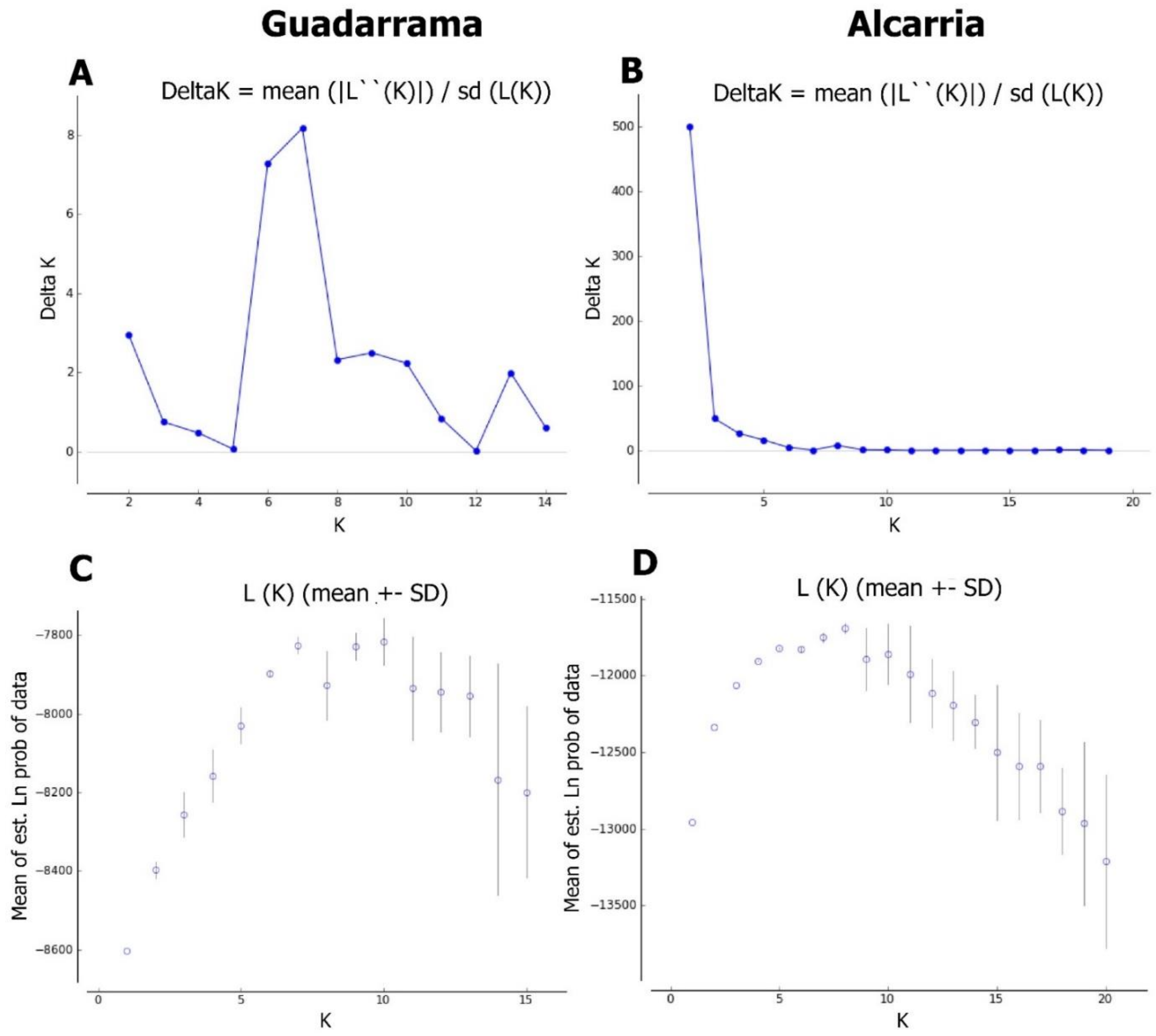

E

K	Reps	Mean LnP(K)	Stdev LnP(K)	Ln'(K)	|Ln''(K)|	Delta K
1	10	-8,602.7	0.4	NA	NA	NA
2	10	-8,397.8	21.7	204.9	64.0	2.9
3	10	-8,256.9	57.0	140.9	42.4	0.7
4	10	-8,158.4	65.3	98.5	30.5	0.5
5	10	-8,029.4	46.1	129.0	2.7	0.1
6	10	-7,897.7	8.2	131.7	59.7	7.3
7	10	-7,825.7	21.4	72.0	174.7	8.2
8	10	-7,928.3	87.1	-102.6	201.9	2.3
9	10	-7,829.0	34.9	99.3	87.1	2.5
10	10	-7,816.7	58.8	12.3	131.2	2.2
11	10	-7,935.7	131.6	-119.0	109.8	0.8
12	10	-7,944.9	100.7	-9.2	1.4	0.0
13	10	-7,955.5	101.3	-10.6	201.0	2.0
14	10	-8,167.2	293.4	-211.7	179.0	0.6
15	10	-8,199.9	217.5	-32.7	NA	NA

F

K	Reps	Mean LnP(K)	Stdev LnP(K)	Ln'(K)	|Ln''(K)|	Delta K
1	10	-12,958.5	0.4	NA	NA	NA
2	10	-12,336.1	0.7	622.4	348.4	499.5
3	10	-12,062.1	2.4	274.0	115.8	49.2
4	10	-11,903.9	2.9	158.2	75.8	26.3
5	10	-11,821.6	5.6	82.4	90.4	16.1
6	10	-11,829.5	18.2	-8.0	87.9	4.8
7	10	-11,749.6	29.5	80.0	20.7	0.7
8	10	-11,690.4	32.9	59.2	260.2	7.9
9	10	-11,891.3	201.0	-200.9	232.3	1.2
10	10	-11,859.9	197.0	31.4	161.5	0.8
11	10	-11,990.0	314.7	-130.1	6.0	0.0
12	10	-12,114.2	220.8	-124.2	43.0	0.2
13	10	-12,195.4	224.4	-81.2	24.7	0.1
14	10	-12,301.3	173.9	-105.9	96.0	0.6
15	10	-12,503.2	443.1	-201.9	111.9	0.3
16	10	-12,593.2	347.3	-90.0	90.2	0.3
17	10	-12,593.0	301.4	0.2	291.3	1.0
18	10	-12,884.1	281.0	-291.1	209.2	0.7
19	10	-12,966.1	531.7	-82.0	165.7	0.3
20	10	-13,213.8	566.1	-247.7	NA	NA

Supplementary Figure S3. Plot of pairwise geographic Euclidean distances (km) vs genetic distances (G'_{ST}) in both study areas.

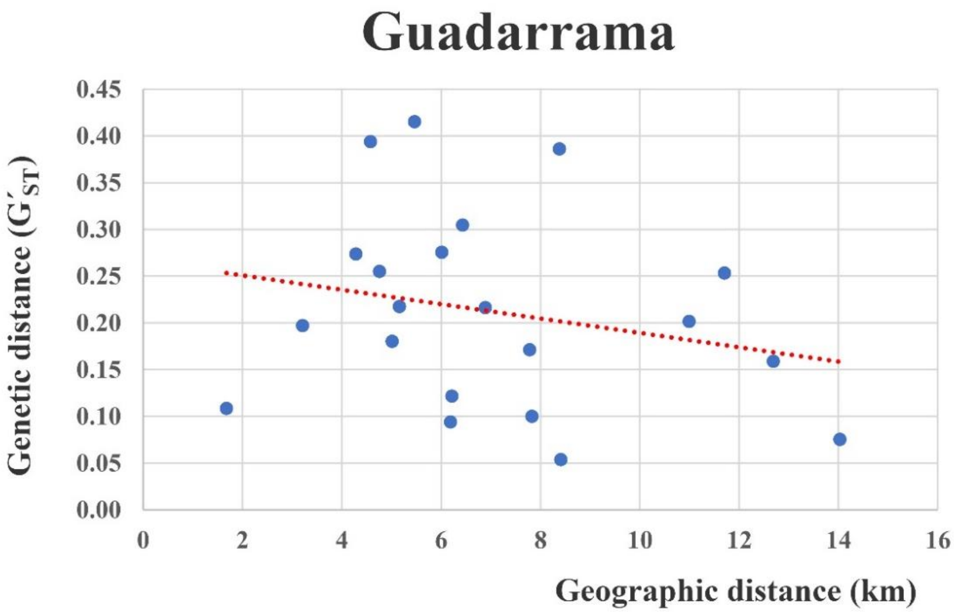

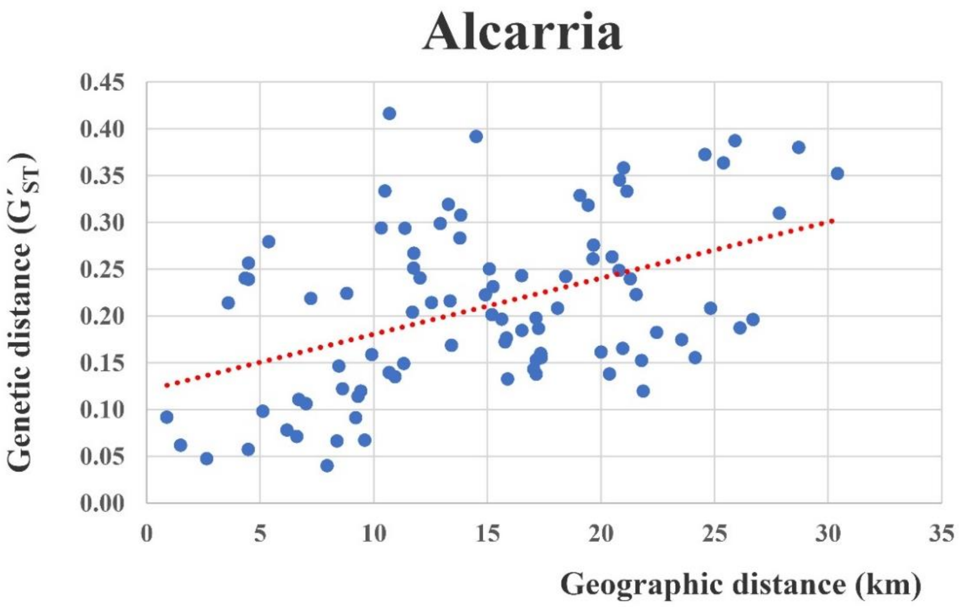

Supplement: Supplementary file 1 — Supplementary Material 1 [file 41598_2026_36452_MOESM1_ESM.pdf]
